# Quantum Chemical Microsolvation via Free Energy–Based Solvation Site Identification

**DOI:** 10.1021/acs.jctc.6c00994

**Published:** 2026-09-08

**Authors:** Lukas B. Magenheim, Maren Podewitz

**Affiliations:** Institute of Materials Chemistry, 27259 TU Wien, Getreidemarkt 9, 1060 Vienna, Austria

## Abstract

In quantum chemical descriptions of solutions, solvents are treated either implicitly, accounting only for bulk properties, or explicitly by including solvent molecules in the calculations. Although explicit treatments are more accurate, they are often computationally prohibitive. As a cost-effective alternative, microsolvation models have emerged that include only a limited number of solvent molecules to capture the most relevant solute–solvent interactions. However, determining their optimal number, positions, and orientations remains a challenge. Existing approaches typically either build up solvent clusters in a stepwise, order-dependent manner or select solvent molecules based on geometric criteria without directly quantifying interaction strength. Here, we introduce the Free Energy Based Identification of Solvation Sites (FEBISS) 2, a revised microsolvation protocol based on Grid Inhomogeneous Solvation Theory (GIST) applied to molecular dynamics trajectories. The method identifies thermodynamically favorable solvation sites from Free Energy contributions. It automatically places, orients, and ranks solvent molecules by interaction strength, using a statistically averaged, order-independent orientation procedure that is applicable to arbitrary rigid solvents rather than water alone. This approach provides a physically grounded criterion for selecting the number of solvent molecules in microsolvated clusters. We demonstrate the applicability of the approach across a range of systems, from small organic molecules to transition-metal catalysts, and for various solvents. We show that explicit microsolvation affects the electronic structure even in systems traditionally considered weakly solvent-sensitive, highlighting FEBISS 2 as a practical methodology to set up physically meaningful microsolvation clusters for quantum chemical studies.

## Graphical abstract

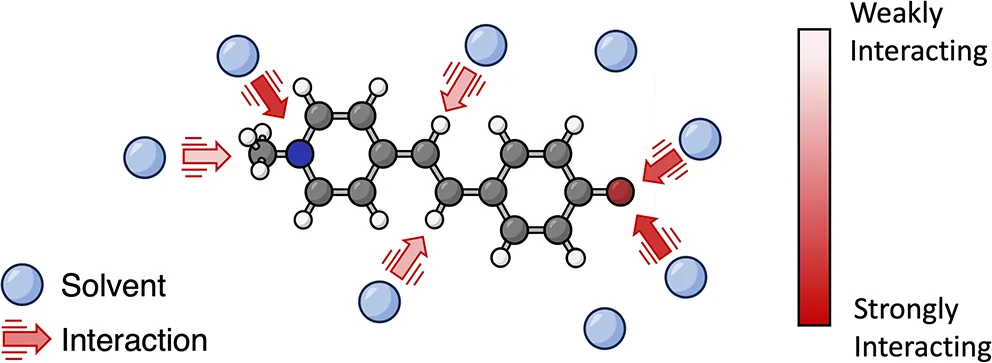

## Introduction

Many chemical processes in nature and modern chemistry occur in solution. Solvents act as mediating agents not only on a macroscopic level, by maintaining properties, such as temperature and pressure, but also on a microscopic level, where they can directly influence molecular interactions and even participate in reactions.^1^ A detailed understanding of these processes requires careful consideration of how the solvent environment affects the physicochemical properties of dissolved species. Computational chemistry plays a key role in this context, providing an atomistic perspective to unravel the origins of complex solvent effects on chemical reactivity.^2,3^

In quantum chemical calculations, solvent effects are often taken into account by treating the solvent as a continuum with a constant dielectric permittivity *ε* surrounding the compound of interest which is typically embedded in a molecular shaped cavity (Figure 1, left). Such models are straightforward to use at marginally increased computational costs but at significantly higher accuracy. Consequently, they have been widely adopted and the most popular ones are the Polarizable Continuum Model (PCM) first described by Tomasi and co-workers^4,5^ as well as the closely related Conductor-like Screening Model (COSMO).^6,7^ A comprehensive overview of the various polarizable continuum models (PCM) and their relationship to COSMO is given by Herbert.^8^ In addition, approaches such as COSMO-RS^9,10^ and SMD^11^ extend these models by incorporating non-electrostatic interactions between the solute cavity and the surrounding continuum.

**Figure 1:**
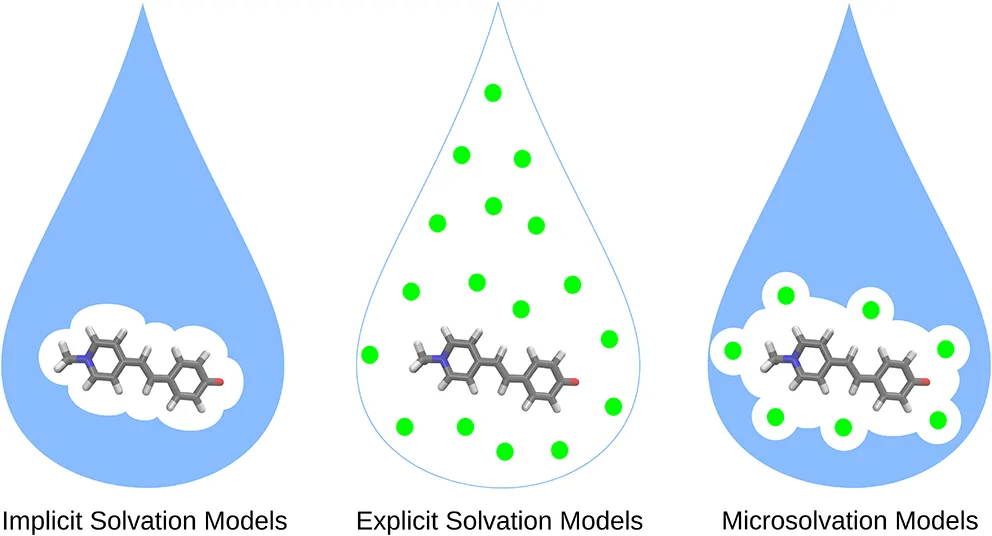
Different approaches to model solvation effects.

Recent developments have further advanced implicit solvation methods. For example, the Riniker group^12,13^ combined classical molecular dynamics simulations with machine-learning to parameterize implicit solvation models for quantum mechanical applications. While promising, their applicability to homogeneous catalysis and to the systematic exploration of reaction mechanisms remains to be established. Despite these improvements, standard implicit solvation models still struggle to describe strong, directional solute–solvent interactions such as hydrogen bonding.^14^ As a result, structural motifs governed by these interactions are often inadequately captured.^15^ In certain cases, implicit models can even fail qualitatively. For instance, we have shown that explicit solvation is essential to correctly describe the conformational space and to preserve the structure and function of a macrocyclic calix[8]­arene-based copper catalyst.^16^

Including solvent molecules explicitly at the quantum chemical level (Figure 1, middle), however, increases the complexity of the chemical system by requiring conformational sampling through ab initio Molecular Dynamics simulations. Such simulations are very expensive and not used on a routine basis for mechanistic explorations of medium to large systems.^17^ While machine-learned interatomic potentials hold great promise to speed up calculation time,^18,19^ accurate training is still non-trivial despite the rise of foundation models.^20,21^ Quantum mechanics/molecular mechanics (QM/MM) methods reduce computational costs by treating only the chemically important region quantum mechanically, while describing the surrounding environment with molecular mechanics. However, in the widely used electrostatic embedding approach, the solvent influences the quantum calculation only through fixed molecular mechanics partial charges.^22,23^

As an intermediate between implicit and explicit solvation models, the reference site interaction model (RISM) has emerged.^24–26^ While the 1D-RISM variant only accounts for the intersite distance, 3D-RISM considers three-dimensional averaged solvent distributions.^27–30^ We would like to emphasize that these models output continuous distribution functions rather than explicit solute–solvent interactions. Post-processing tools, such as Placevent,^31^ GASol,^32^ and Solv-eze,^33^ exist that convert these continuous distributions to explicit solvent positions but they are all limited to water.

A hybrid model that directly takes explicit solute–solvent interactions into account is the microsolvation or cluster-continuum approach:^14^ Here, only *the most important* solvent molecules are explicitly included in the quantum chemical description while the bulk is treated implicitly (Figure 1, right), thus maintaining a feasible computational workload. We would like to point out here that just increasing the number of explicit solvent molecules in the quantum chemical calculation does not necessarily improve the results^34^ but a meaningful selection of explicit solvents, for example based on solute-solvent interaction strength, is key. Algorithms which help in this task can be roughly categorized into two groups: the top-down and the bottom-up approaches.^15,35^ The latter ones start with the bare solute and gradually build up the solvation shells. For example, Simm et al.^36^ introduced an algorithm that does this in a stochastic manner ensuring the formation of homogeneous solvation shells. Another approach is pursued by Spicher et al.^37^ in the Quantum Cluster Growth algorithm where the geometry of the resulting solute-solvent cluster is optimized after each addition of a solvent molecule. ORCA 6 ^38^ also features the possibility of generating microsolvated clusters with the SOLVATOR functionality which either places the solvent molecules stochastically or via a docking algorithm. A common feature of these bottom-up strategies is the sequential addition of solvent molecules, with the cluster typically being optimized before the next solvent molecule is placed. Consequently, the placement and orientation of each molecule can influence, and be influenced by, those already present, introducing an order dependence that is inherent to the stepwise construction process.

Top-down approaches on the other hand start from a dynamic description of the solution, usually by means of Molecular Dynamics (MD) simulations, which have the advantage to consider only reliable solution-phase conformations. One possibility to arrive at microsolvated structures is to extract snapshots from the resulting trajectories. This approach is followed for instance in AutoSolvate,^39^ recently extended to work with user friendly large language models^40^ and in Solvate Suite.^41^ A shortcoming of this strategy is that snapshot and solvent molecule selection is typically based on geometric criteria, such as solute-solvent distance or shape, rather than on the underlying interaction strength. The solute-solvent interaction strengths and thermodynamic quantities are then only a posteriori assessed for the selected clusters, rather than informing the selection itself.

In contrast, our group has developed the Free Energy Based Identification of Solvation Sites algorithm (FEBISS),^35^ where the placement of solvent molecules is based on thermodynamic quantities. In this top-down approach, full condensed-phase MD simulations are used as a starting point. By employing Grid Inhomogeneous Solvation Theory (GIST) on the resulting trajectory,^42^ we obtain information on the spatial distribution of the Free Energy of Solvation around the solute. When also analyzing the solvent density around the solute, this approach allows for the identification of areas of most favorable solute-solvent interactions, and subsequently for the placement of solvent molecules (central oxygen atom) around the solute. Density-based solvent placement itself is not unique to FEBISS. Placevent^31^ introduced a related approach to convert 3D-RISM continuous distributions to explicit solvent positions, and similar oxygen-only, density-based placement strategies have since been developed (e.g., GAsol^32^). However, these methods do not account for molecular orientation. By contrast, FEBISS explicitly resolves solvent orientations, which is particularly relevant for multi-site solvents. Solvent orientations were obtained by locating maxima in the elemental density for the remaining hydrogen atoms, given the geometry constraints. Because this orientation is derived as a statistical average over the complete condensed-phase trajectory, rather than assembled molecule by molecule, it is intrinsically independent of placement order and fundamentally different from the iterative bottom-up strategies described above. However, relying on elemental densities to determine molecular orientation, as in our initial FEBISS implementation,^35^ has significant shortcomings: the position of the second hydrogen atom could not always be uniquely determined from its elemental density alone.

Herein we describe a completely revised version of the Free Energy Based Identification of Solvation Sites 2 (FEBISS 2), which is now fit to deal with arbitrary rigid solvents, providing a general and transferable framework for solvent selection, interaction quantification, and orientation determination across diverse solvent environments, including those central to reaction mechanism exploration in organic and organometallic chemistry. Key to this extension are (i) a new, rigorous approach to finding statistically averaged orientations of the placed solvent molecules utilizing a solvent substructure rather than elemental densities, and therefore being applicable across a broad range of solvent types; (ii) the possibility to use the center-of-mass or a predefined central atom of the solvent molecule for placement; (iii) a user-friendly interface that allows routine use of FEBISS 2 for study of microsolvation in reactivity of organic and organometallic compounds; and (iv) a set of ten commonly used, pre-parameterized solvents, enabling immediate, out-of-the-box application of FEBISS 2 to the most relevant organic and organometallic reaction media without additional setup.

In the first section of this work, we give a minimal introduction to GIST. This part is followed by a short overview of the algorithm and a detailed description of the FEBISS 2 computational workflow. Subsequently, we discuss the applicability of our methodology at a number of different solutes, including small (organic) molecules, like urea, (+)-borneol and Brooker’s merocyanine dye in various solvents. Lastly, we investigate the effect of microsolvation on a molybdenum imido alkylidene N-heterocyclic carbene Schrock-type catalyst for olefin metathesis in dichloromethane.

## Grid Inhomogeneous Solvation Theory

We give a brief outline of the Grid Inhomogeneous Solvation Theory. For a more rigorous review of this topic the reader is referred to a selection of papers.^42–44^ Furthermore, we follow here the formalism of the current implementation of GIST in CPPTRAJ^45,46^ developed by Waibl et al.,^47^ an extension to the original implementation.^48^

The Inhomogeneous (Fluid) Solvation Theory (IST) devised by Lazaridis^43,44,49,50^ allows the spatially resolved evaluation of the Free Energy of Solvation of a solute using MD simulations. In IST, the solute, denoted here as *u*, is regarded as a particle fixed at the origin. It gives rise to an external field which the surrounding solvent molecules, denoted as *v*, experience. The Free Energy of Solvation is then given as

1 $$\Delta G_{\mathrm{solv}}=\Delta E_{\mathrm{solv}}-T\Delta S_{\mathrm{solv}}=\Delta E_{\mathrm{uv}}+\Delta E_{\mathrm{vv}}-T\Delta S_{\mathrm{uv}}-T\Delta S_{\mathrm{vv}}$$

Here, the internal energy and entropy of solvation, Δ*E*_solv_ and Δ*S*_solv_, are decomposed into contributions from solute–solvent interactions, Δ*E*_uv_, Δ*S*_uv_, and the solvent “reorganizational”^43^ energy and entropy, denoted as Δ*E*_vv_ and Δ*S*_vv_, which arises due to the presence of the solute molecule. *T* is the absolute temperature.

The Δ*S*_uv_ term can be split further into an orientational and translational term

2 $$\Delta S_{\mathrm{uv}}=\Delta S_{\mathrm{uv}}^{\mathrm{trans}}+\Delta S_{\mathrm{uv}}^{\mathrm{or}}$$

Expressions for these terms were first formulated by means of continuous integral equations over space, which are difficult to evaluate. Grid Inhomogeneous Solvation Theory,^42^ approximates these integrals by dividing the solvation box into discrete voxels *k* and calculating the Δ*G*_solv_ contributions per voxel. Using eqs 1 and ([Disp-formula eq2]), the contribution of the voxel *k* to the Free Energy of solvation is given as

3 $$\Delta G_{\mathrm{solv},k}=\Delta E_{\mathrm{uv},k}+\Delta E_{\mathrm{vv},k}-T\Delta S_{\mathrm{uv},k}^{\mathrm{trans}}-T\Delta S_{\mathrm{uv},k}^{\mathrm{or}}-T\Delta S_{\mathrm{vv},k}$$

The energy terms, Δ*E*_uv,k_ and Δ*E*_vv,k_, are easily calculated using pair-wise atomic potentials defined by the force field of the simulation.^42,47^

The translational solute-solvent entropy Δ*S*_uv,*k*_^trans^ is approximately calculated by means of

4 $$\Delta S_{\mathrm{uv},k}^{\mathrm{trans}}\approx R\left(\gamma +\frac{1}{N_{k}}\sum_{k}\ln\frac{N_{f}\rho^{0}4\pi d_{\mathrm{trans},k}^{3}}{3}\right)$$

where *R* is the universal gas constant, γ the Euler–Mascharoni number, and *N_k_* the number of solvent molecules that were found in voxel *k* across all *N_f_* frames. ρ^0^ denotes the reference density of the bulk solvent and *d*_trans,*k*_ is the smallest distance between two solvent molecule in voxel *k* using a nearest-neighbor approach.^51,52^

Similarly, the Δ*S*_uv,*k*_^or^ term can be approximated as

5 $$\Delta S_{\mathrm{uv},k}^{\mathrm{or}}\approx R\left(\gamma +\frac{1}{N_{k}}\sum_{k}\ln\frac{N_{f}\Delta \omega_{k}^{3}}{6\pi }\right)$$

Here, Δω*_k_* is the distance between the orientations of two solvent molecules expressed as unit quaternions. The Δ*S*_vv_ term is completely neglected in this formalism. The main drawback of GIST is the restriction upon the solute to remain conformationally rigid. For flexible solutes, a separate MD simulation with subsequent GIST analysis needs to be carried out for each conformation.

## Computational Protocol

The overall workflow of our microsolvation approach is shown in Figure 2 and consists of the following steps: conformational sampling of the solute (optional), classical MD simulation of the restrained solute, GIST analysis, solvent placement and Free Energy assignment, ranking and selection of solvents, determination of average solvent orientations, and finally the generation of microsolvated clusters. A further post-processing step is optional.

**Figure 2:**
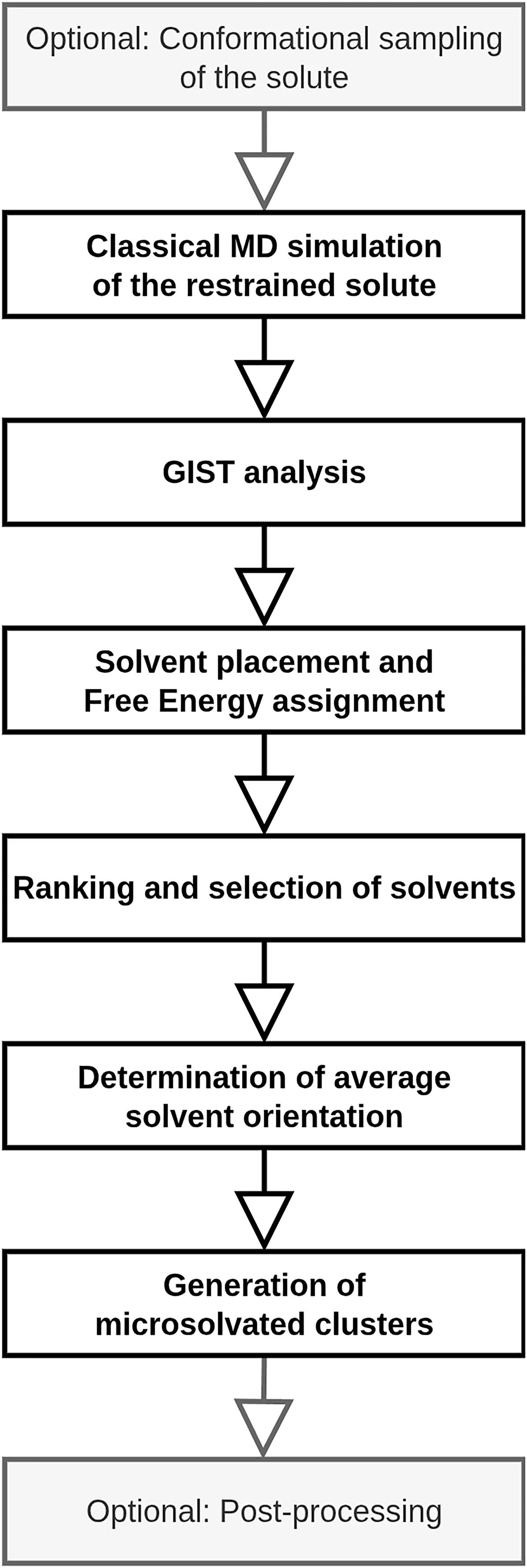
Workflow of FEBISS 2 protocol.

### Classical MD Simulation of the Restrained Solute

We first subject the solute to a classical MD simulation which can be performed by any software as long as the trajectory output can be read by CPPTRAJ.^45,46^ Therefore, the solute is restrained at the origin as required for subsequent GIST analysis. However, this also implies that only one conformation can be analyzed per run. For solutes that feature multiple conformational degrees of freedom, a conformer sampling can be performed beforehand but a restrained MD simulation and subsequent GIST analysis have to be performed for each conformer.

### GIST Analysis

We subject the resulting simulation trajectory to a GIST analysis on a user-defined subvolume of the simulation space, which contains the solute and the solvation region of interest. Typically, we chose a 30 × 30 × 30 Å^3^ cubic box. This box gets further divided into separate voxels *k* for which we obtain the thermodynamic quantities Δ*E*_uv,*k*_, Δ*E*_vv,*k*_, Δ*S*_uv, *k*_^or^, Δ*S*_uv, *k*_^trans^ which readily give Δ*G*_solv,*k*_. In addition, we also acquire data on the solvent density for each voxel *k*, and the orientations of the solvent molecules in each simulation, the latter of which is required to calculate the Δ*S*_uv,*k*_^or^.

To assess the orientation of each solvent molecule, we conveniently define a central point of the solvent, either an atom or the center-of-mass (COM) as *P*_0_ and determine two additional points/atoms as *P*_1_, *P*_2_. These three points uniquely determine the orientation of each solvent molecule in each frame of our MD simulations. For Δ*S*_uv, *k*_^or^ the orientational distance between pairs of solvent molecules is calculated using quaternions.

The voxel occupancy θ_k_ is straightforwardly determined by counting the appearances of *P*_0_ of any solvent molecule and dividing it by the number of frames of the simulation trajectory. Consequently, a voxel occupancy of one would be obtained if a voxel is occupied by a solvent molecule in each frame of the simulation. A solvent molecule is considered within a voxel if the central point *P*_0_ is found within the voxel’s borders, with a standard voxel size of 0.5 × 0.5 × 0.5 Å^3^.

### Solvent Placement and Free Energy Assignment

The algorithm places the central point *P*_0_ of the solvent molecule in the middle of the voxel with the highest occupancy (Figure 3(a)). However, as no voxel typically has an occupancy of one, occupancies are successively accumulated from the central voxel and neighboring voxel shells until a cumulative occupancy of one is reached. If the inclusion of the outermost shell would exceed this value, its occupancies are scaled proportionally so that the total occupancy equals exactly one. The Free Energy of the placed solvent molecule is then calculated as the occupancy-weighted sum of the contributions of each voxel, given by

6 $$\Delta G_{\mathrm{solv}}=\sum_{i}\sum_{k_{i}}^{k_{\max,i}}\theta_{k_{i}}\Delta G_{\mathrm{solv},k_{i}}$$

Here θ_*k_i_*_ is the voxel occupancy of voxel *k*_*i*_ of shell *i*, and Δ*G*_solv,*k_i_*_ is the Free Energy of voxel *k*_*i*_. The occupancies of the assigned voxels are subsequently set to zero (Figure 3(b)).

**Figure 3:**
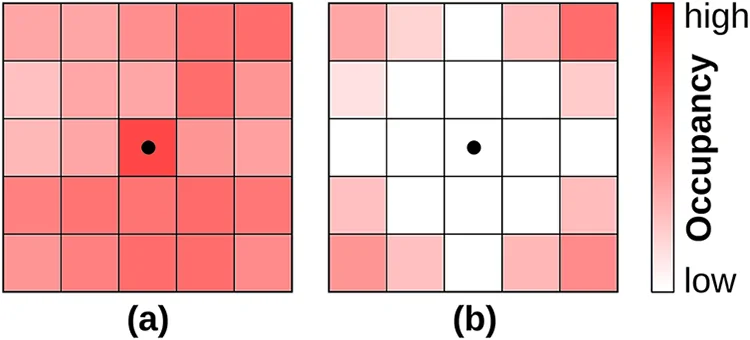
(a) The central voxel was found to have the highest voxel occupancy, thus the central point *P*_0_ of a solvent molecule was placed in its center, stylized as a black dot. (b) After determining the Free Energy of the placed solvent molecule, the algorithm subtracts voxel occupancy starting from the central voxel and including neighboring shells until an occupancy of one is reached. The outermost shell is scaled to exactly yield an occupancy of one.

### Ranking and Selection of Solvents

Our program ranks the placed solvent molecules according to their Free Energy and displays them as an interactive bar plot.

Each bar represents a particular solvent molecule which can be clicked for selection. Based on the Free Energy, considerations derived from radial distribution functions (RDFs), and a color coding based on the distance between solute and solvent, the user may select a number of solvent molecules. The color coding is thereby based on the closest distance between any atom of the solvent to any atom of the solute. The distance thresholds depend on the system under investigation and were set manually based on the size of the solvent molecules. They are therefore intended only as a rough guidance when inspecting the spatial solvent distribution rather than a rigorously defined selection criterion.

### Determination of Average Solvent Orientations

Upon selecting the desired solvent molecules, the program calculates the average orientation of the placed molecules. Each solvent molecule is described by three points, the center-of-mass (COM) or a user-defined central atom and two additional points (atoms) that uniquely define the orientation. Please note that COM placement is the default. However, in classical MD simulations atoms are numbered, hence, chemically equivalent atoms are distinguishable. As a consequence, chemically equivalent orientations linked by symmetry operations give rise to different quaternions and this must be addressed when calculating the average orientation.

Taking the example of chloroform, we first define an arbitrary reference orientation (see Figure 4) with the quaternion *q*_ref_, as well as the symmetry equivalent orientations *q*_1_ and *q*_2_. For each solvent molecule in each frame, we determine to which of these symmetry equivalent orientations it has the smallest distance to, e.g., to *q*_1_. Subsequently, by applying the unitary rotation, here *q*_a_, we map the solvent to the reference orientation and calculate an average configuration according to the maximization procedure given by Markley et al.^53^ More information is provided in the Supporting Information.

**Figure 4:**
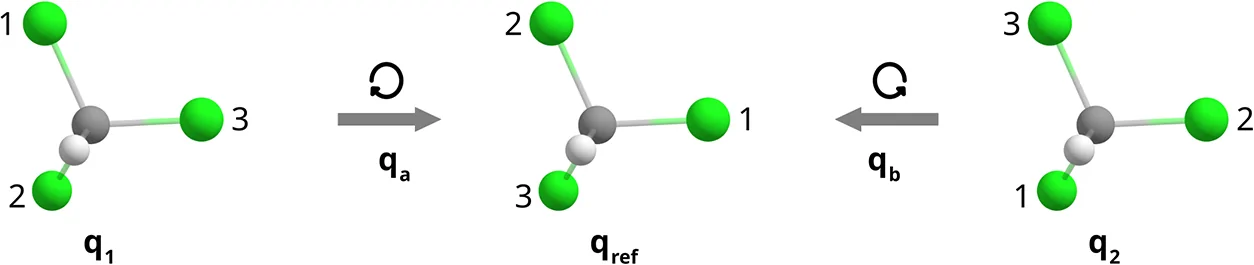
In MD simulations all atoms are uniquely labeled. Taking chloroform as an example, which has *C*_3*v*_ symmetry, all chlorine atoms are chemically equivalent, and the orientations *q*_1_, *q*_ref_, and *q*_2_ are therefore equivalent as well. By applying the rotations *q*_a_ and *q*_b_, each of these orientations can be mapped onto the reference orientation *q*_ref_.

### Generation of Microsolvated Clusters

The resulting microsolvated structure is generated as a PDB file that can be used for further analysis and post-processing.

### Post-Processing

As the placed solvent molecules are in averaged orientation, the microsolvated cluster may further be optimized so that an energy minimum is obtained. The fastest option is a QM/MM minimization, where the solute is treated at a quantum mechanics level and the solvent at a molecular mechanics level. Of course, the whole cluster can also be minimized at full quantum level. The choice of quantum method depends on the system under investigation, ranging from efficient semi-empirical approaches such as GFN2-xTB^54^ or g-xTB^55^ to full density functional theory (DFT) or even wave function theory. For a final assessment of solvent effects, a full quantum calculation on the complete cluster is required to capture the influence of explicit solvent beyond simple electrostatic interactions.

More information on the implementation and usage of FEBISS is detailed in the Supporting Information.

## Computational Methodology

### Molecular Dynamics Simulation

Prior to any MD simulation, force field parameters for all solutes needed to be generated. In order to do so, quantum chemical structure optimizations were performed with ORCA 5 ^38^ at DFT level. For urea, the B3LYP density functional^56–58^ was used in conjunction with the def2-TZVP basis set^59^ and empirical dispersion corrections of the D4 type, which include tight binding partial charges.^60,61^ A conductor-like polarizable continuum model (C-PCM)^62^ was used to approximately describe the implicit environment. For urea, a dielectric constant of *ϵ* = 80.4 and a refractive index *n* = 1.33 describing bulk water was employed. The same basis set and functional was used for the optimization of both (+)-borneol and Brooker’s merocyanine dye. D3BJ^63^ dispersion correction was used for (+)-borneol, D4 in the case of Brooker’s merocyanine dye. Also, C-PCM with the respective dielectric constant of methanol (ϵ = 32.63, *n* = 1.329), chloroform (ϵ = 4.90, *n* = 1.450), dimethyl sulfoxide (DMSO) (ϵ = 47.2, *n* = 1.479), and tetrachloromethane (ϵ = 2.24, *n* = 1.344) were used for optimizations of Brooker’s merocyanine dye.

For consistency with previous studies, the molybdenum imido alkylidene N-heterocyclic carbene catalysts was optimized with BP86 ^56,64^ /def2-SVP^59^/D3BJ.^63^ To model the implicit dichloromethane permittivity parameters of ϵ = 9.08 and *n* = 1.424 were used.

The quantum chemically optimized structures were parametrized with the antechamber module of AmberTools,^65^ which employs the Generalized Amber Force Field (GAFF).^66^ Metal–ligand parameters for the Mo complex were obtained using the bonded model approach,^67^ as implemented in Multiwfn 3.8,^68^ where force constants are derived from analysis of the Hessian.

Atomic charges for all species were determined via the restrained electrostatic potential (RESP) protocol,^69^ also implemented in Multiwfn 3.8. An orthorhombic box was then constructed and filled with the corresponding solvent molecules. Box sizes range from roughly 45 × 45 × 45 for urea to Å^3^ to roughly 60 × 60 × 60 Å^3^ for Brooker’s dye in several solvents. The average box size across the entire trajectory is given in the Supporting Information Table S01. For Brooker’s dye, separate geometry optimizations and parametrizations were carried out for each solvent to ensure a consistent computational approach for each solvent. Respective force field parameters of all solutes are listed in the Appendix of the Supporting Information.

The equilibration was carried out using an adapted protocol devised by Wallnoefer,^70^ which includes multiple heating and cooling steps to ensure the system has reached a steady state. To streamline this procedure, we used our PyConSolv tool that automates all the previously described steps with minimal user intervention by interfacing to the respective codes.^71^ MD simulations were carried out with Amber 24, or with Amber 20 in the case of the molybdenum catalyst.

Note that performing a GIST analysis on an MD trajectory requires a restrained simulation. For rigid molecules, the solute is restrained to its dominant conformation. For flexible molecules, an initial MD simulation in explicit solvent must first be carried out to identify relevant conformers. This step can conveniently be done with PyConSolv, which automates this workflow. For each identified conformer, a separate restrained simulation must then be performed to enable the GIST analysis. This approach was chosen for the conformationally flexible molybdenum imido alkylidene N-heterocyclic carbene catalyst. From the conformational analysis, a representative structure was selected as input for the restrained simulation as needed for GIST.

For the pre-parameterized solvents (methanol, ethanol, chloroform, acetonitrile, acetone, DMSO, benzene, dichloromethane, tetrachloromethane, and water), no additional steps are required. For other solvents, an additional MD simulation of the pure solvent box must be performed to determine the reference solvent density and the reference solvent–solvent interaction energy (Δ*E*_vv_). These quantities can be supplied to the FEBISS 2 analysis either through the interactive interface or via a YAML settings file. Finally, the solvent substructure used to define the solvent orientation must be specified.

The total length of the GIST production NpT simulation was set to 100 ns, which was considered to be long enough to obtain converged results for most solvents.^47^ An integration time step of 1 fs was chosen; every 10 ps a snapshot was taken and stored, totaling 10,000 frames that were used for further analysis. The solute was restrained in a harmonic potential with a force constant of at least 100 kcal mol^–1^ Å^–2^. The temperature was kept constant at 300 K using a Langevin thermostat^72^ with a collision frequency of 2 ps^–1^. The pressure was set to 1 bar, using a Berendsen barostat^73^ with a pressure relaxation time of 2 ps and isotropic position scaling.

### GIST Analysis and Solvent Placement

The GIST analysis and subsequent placement of solvent molecules were carried out using FEBISS 2.0, which interfaces with CPPTRAJ for GIST calculations. A modified version of CPPTRAJ v6.29.1 ^45,46^ was employed, including extended functionality for defining the solvent central point (used for placement), as well as the capability to output quaternion data required to determine the average orientation of solvent molecules. The GIST analysis is typically performed on a 30 × 30 × 30 Å^3^ subvolume of the simulation box for each investigated system. We defined a cubic grid centered at the geometric center of the solute consisting of 60 × 60 × 60 voxels with a voxel edge length of 0.5 Å in each dimension. RDFs were additionally computed with CPPTRAJ for selected solute atoms. All 10,000 frames of the production run were subjected to the GIST analysis. If not explicitly noted otherwise, the COM is invoked for the solvent placement. Although the implemented algorithm is applicable to any rigid solvent, pre-parameterized solvents are provided for methanol, ethanol, chloroform, acetonitrile, acetone, DMSO, benzene, dichloromethane, tetrachloromethane, and water.

### Post-Processing

The microsolvated clusters of urea and (+)-borneol were analyzed as generated with FEBISS 2 without any further post-processing. In contrast, we subjected the Brooker’s merocyanine dye microsolvated in methanol and chloroform to a DFT optimization using B3LYP/def2-TZVP/D4 in the respective implicit C-PCM solvent model as implemented in ORCA 5.0.4.^38^ The resulting microsolvated molybdenum imido alkylidene N-heterocyclic catalyst–dichloromethane cluster was optimized using QM/MM instead. Thereby, we chose the atoms of the molybdenum catalyst to be treated quantum mechanically using BP86/def2-SVP/D3BJ, the solvent molecules were treated on the molecular mechanics instead, reusing the force field parameters from the simulation. For final evaluation of bond order indices, a full DFT single point (BP86/def2-SVP/D3BJ/C-PCM) was performed on the entire microsolvated cluster.

For sake of comparison, we removed the explicitly given solvent molecules and performed a single point calculation in the gas phase and an implicit C-PCM using the same methodology. The resulting wave functions were analyzed by determining Mayer bond order indices^74–76^ as implemented in ORCA 5.0.4.

## Results

To give an overview over the applicability and scope of our new FEBISS 2 protocol, we investigated a number of different systems: (1) urea in water because we previously studied this system with our initial FEBISS implementation, (2) (+)-borneol in DMSO, (3) Brooker’s merocyanine dye, which is a system that is soluble in a range of different solvents and which we investigated in methanol, chloroform, DMSO, and tetrachloromethane, and (4) a typical transition-metal catalyst, here a molybdenum imido alkylidene N-heterocyclic carbene catalyst in dichloromethane, capable of olefin metathesis, shedding light on the ongoing discussion of the role of explicit solvent in homogeneous catalysis.^77,78^

### Urea in Water

We started our evaluation by investigating urea in water, which we have studied before.^35^ Following the outlined FEBISS 2 computational protocol, we ran a restrained solute MD simulation, performed a GIST analysis, and placed solvents at positions of high solvent density. Each placed solvent was assigned its GIST free energy and its averaged orientation was obtained by analysis of each trajectory frame. The solvents were ranked by their free energy in a bar plot, from which the microsolvated clusters were generated by selecting a number of solvent molecules (Figure 5). Our newly implemented methodology allows for the placement of solvent molecules based on the solvent’s center-of-mass (COM), which is the default, as well as on the user-defined central atom of the molecule, in the case of water, the oxygen atom.

**Figure 5:**
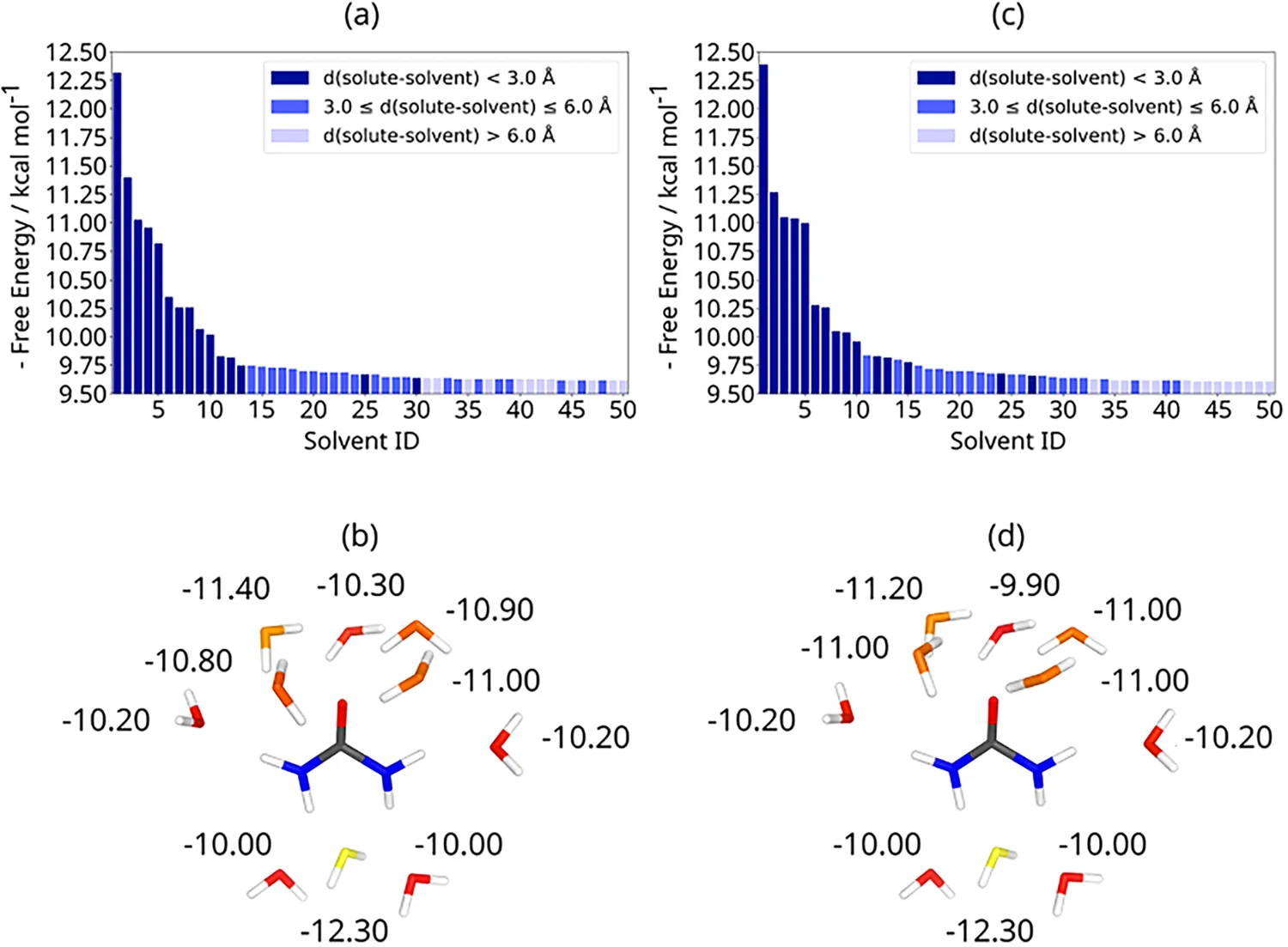
Resulting bar plots obtained with FEBISS 2 workflow and urea-(H_2_O)_10_ cluster structures with the COM as central point (a, b), and oxygen defined as central point (c, d) used for placement, respectively. The solvents in the bar plot are ranked according to their GIST Free Energy are color-coded according to the atom pair-wise closest distance of the solute and solvent with user-defined thresholds, *d* < 3.0 Å, dark blue, 3.0 Å ≤ *d* ≤ 6.0 Å, medium blue, and *d* > 6.0 Å, light blue. The water’s oxygen atoms in (b) and (d) are color-coded from yellow to red according to their assigned Free Energy value, which is given in kcal mol^–1^.

The results of these two placements are depicted in Figure 5(a),(b) (COM) and 5(c),(d) (central oxygen atom), respectively. The bars are color-coded according to the solute-solvent distance, calculated as the closest distance between any pair of solute and solvent atoms. For water, cut-off values of 3.0 Å and 6.0 Å were chosen based on the size and shape of the solvent molecule, serving as a rough guide for the user to assess the distance between solvent and solute. They are not intended as a rigorously defined solvation-shell criterion. Each bar represents a solvent molecule ranked according to the GIST-based Free Energy assigned. Now for choosing an appropriate number of solvent molecules, we primarily look for solvent molecules closest to the solute and pronounced jumps in Free Energy. For urea, we find several such jumps in interaction energy before the bar plot smoothly converges towards the Δ*E*_vv_ value of −9.544 kcal mol^–1^ of neat unperturbed water.

Both bar plots show one very strongly interacting water molecule with −12.3 kcal mol^–1^, located between the two amide hydrogens, showing a bifurcated hydrogen bond. The first ten water molecules are within a solute-solvent distance of less than 3.0 Å. They cluster in a crown-like pattern around the carbonyl moiety and form hydrogen bonds to the amide hydrogens, in agreement with chemical intuition. Comparing absolute values of interaction strength, the results differ less than 0.5 kcal mol^–1^ between the two protocols. Also, the positions of the placed solvent molecules are remarkably similar corroborating the robustness of our method.

In our original FEBISS implementation, the central atom was invoked for initial placement and the orientation of the placed water molecule was determined based on elemental densities. Comparing this ansatz to our new FEBISS 2 implementation that uses quaternion averaged orientations, slight variations in the conformation of the resulting clusters occur. A superposition of both microsolvated structures based on the placement of the oxygen atom can be found in Figure S01.

The choice of voxel size has only a minor impact on solvent placement. While smaller voxel sizes increase spatial resolution, they also increase computational cost and lower voxel occupation numbers, resulting in less robust statistics. Larger voxel sizes decrease computational cost but also lower the spatial resolution (see Supporting Information Section 3 and Figure S04).

Our data is consistent with available 3D-RISM results,^35^ with ab initio MD data^79^ as well as with a bottom-up microsolvation approach.^37^

### (+)-Borneol

As next example, we investigated (+)-borneol in DMSO. This system has been previously investigated by Weirich et al.^80^ by means of vibrational circular dichroism (VCD) spectroscopy and later in greater detail using rotational spectroscopy and computational methods by Pinacho et al.,^81^ highlighting the importance of explicit solvation.

We applied the outlined FEBISS 2 protocol (see Figure 2) protocol to determine strongly interacting solvent molecules for the (+)-borneol in DMSO system, starting from the full condensed-phase description. The resulting bar plot, depicting the (+)-borneol surrounding solvent molecules ranked according to their GIST free energy, is shown in Figure 6, while the atom pair-wise closest solute-solvent distance is color-coded. Cut-off values were chosen based on the size and shape of the solvent molecule to be 4.5 Å and 9.0 Å. Although there are several DMSO molecules in close proximity to the solute (as indicated by the dark blue color), only one solvent strongly interacts with the solute. The microsolvated cluster shows that the oxygen of the DMSO is forming a hydrogen bond with the hydroxyl moiety (Figure 6).

**Figure 6:**
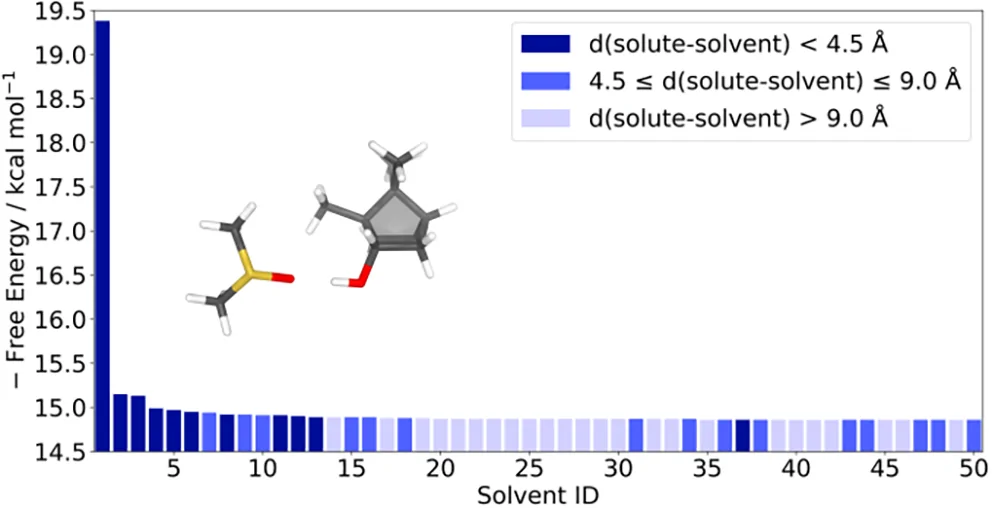
Bar plot of the ranked DMSO molecules obtained with the FEBISS 2 protocol for (+)-borneol, showing one strongly interacting solvent and the resulting cluster. The blue color coding is based on the user-defined atom pair-wise closest distance of solvent and solute. Dark blue: *d* < 4.5 Å, medium blue: 4.5 ≤ *d* ≤ 9.0 Å, light blue: <9.0 Å. Distance thresholds are user-defined and derived from the size of the DMSO molecule.

Our finding is in line with the study of Weirich et al.,^80^ who showed that upon the inclusion of one explicit DMSO molecule, the calculated VCD spectra are in better agreement with experimental data than with implicit solvent models alone.

### Brooker’s Merocyanine Dye

Brooker’s merocyanine dye (*p*-[(*E*)-2-(1-Methyl-1-pyridium-4-yl)­ethenyl]­phenolate)^82^ (Figure 7), short-hand noted as BM, is best known for its solvatochromic behavior^1,83^ and its solubility in a variety of solvents. Here, it was chosen as a test compound for our algorithm due to its rigid structure, its extended apolar π-electron system, and its polar end groups. Its broad solubility makes it an ideal test system for the FEBISS 2 algorithm.

**Figure 7:**
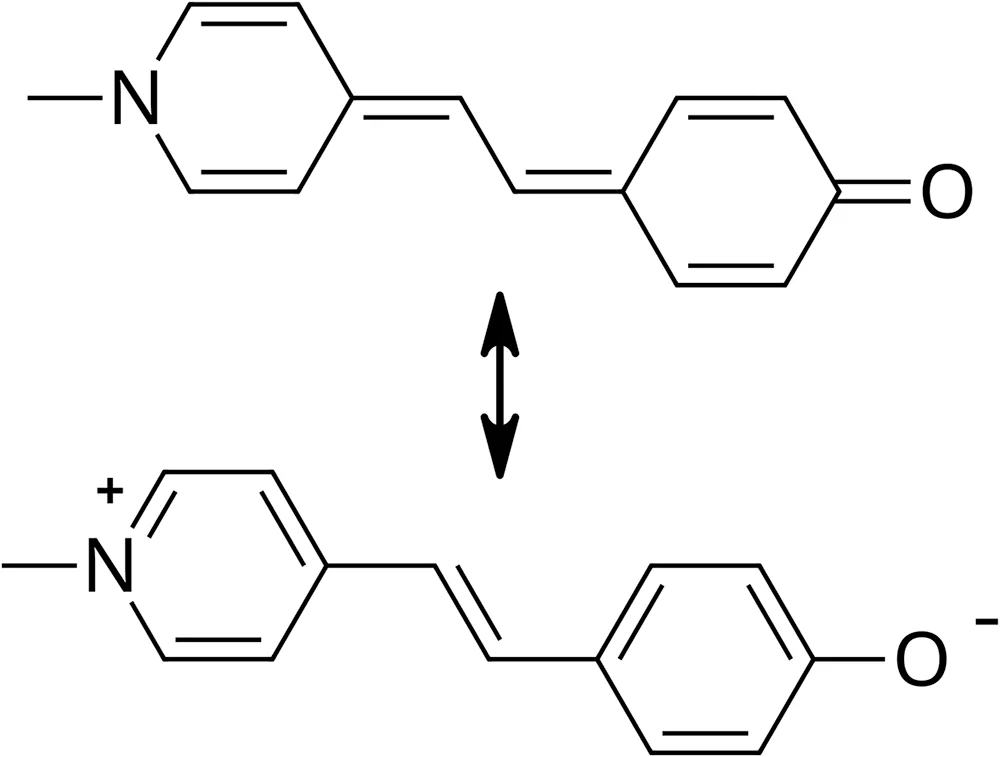
Resonant structures of Brooker’s merocyanine dye (BM).

We investigated solute–solvent interactions of BM in methanol as a polar protic solvent, in DMSO as a polar aprotic solvent, in chloroform as a weakly polar solvent, and in tetrachloromethane as a fully apolar solvent. Note that for each explicit solvent simulation, a separate set of force field parameters was generated.

For methanol (dielectric permittivity ϵ = 32.7), the resulting bar plot obtained after applying the FEBISS 2 workflow (MD simulation, GIST analysis, solvent placement and free energy assignment, ranking, orientation) listing solvent molecules ranked according to their Free Energy is shown in Figure 8(a). The user-defined color coding reveals five methanol molecules within 3.0 Å of the solute, with assigned Free Energy values ranging from −14.2 to −11.7 kcal mol^–1^, see Figure S07. The remaining solvent molecules differ by less than 1 kcal mol^–1^ from the bulk Δ*E*_vv_ value and were therefore not considered.

**Figure 8:**
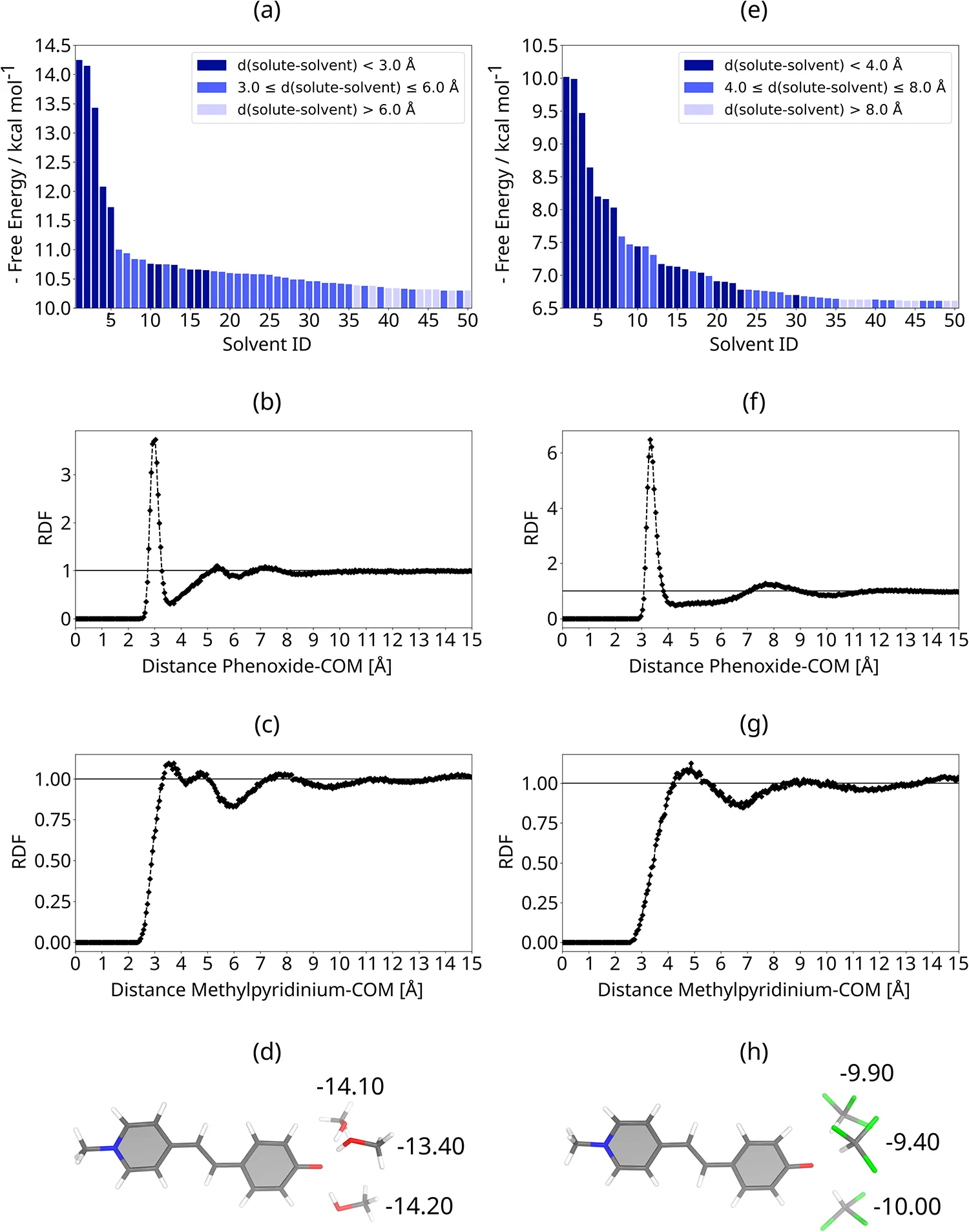
Microsolvation of BM in methanol (MeOH) and chloroform. FEBISS 2 bar plot displaying the solvents ranked according to their Free Energy with color coding indicating user-defined solute-solvent distances as well as RDFs around the phenoxide moiety and the methylpyridinium group for methanol are given in (a–c) and for chloroform in (e–g), respectively. In both cases, the first three solvent molecules were chosen from the bar plot, resulting in the BM-(MeOH)_3_ and BM-(CHCl_3_)_3_ microsolvated structures (d, h). Free energies of placed solvent are given in kcal mol^–1^.

To further assess how many solvent molecules coordinate around specific solute sites, FEBISS 2 supports the calculation and plotting of RDFs. The RDFs of methanol around the phenoxide oxygen atom as well as around the hydrogen atoms of the methyl group are given in Figure 8(b),(c), respectively. By integrating the radial distribution function at the phenoxide-COM up to its first local minimum at around 3.6 Å, we obtain a coordination number of approximately three solvent molecules. Accordingly, selecting only the first three of the five methanol molecules in the bar plot is a reasonable choice for constructing the microsolvated structure shown in Figure 8(d), the microsolvated cluster showing all five methanols is depicted in the Supporting Information, Figure S07. All three solvent molecules form hydrogen bonds with the phenoxide group of BM. The difference between the most strongly interacting methanol and the bulk value is ca. 3.5 kcal mol^–1^. To assess the interaction strength of the formed hydrogen bonds, we optimized the microsolvated cluster and calculated the interaction energy from the two fragments, solute and solvent, respectively. It amounts to −23.3 kcal mol^–1^.

Next, we investigated BM in chloroform with the resulting bar plot being depicted in Figure 8(e). While the interacting energy shows a more gradual decrease compared to methanol and the energy difference between the most strongly interacting solvent and the bulk value is with 3.3 kcal mol^–1^ slightly smaller, more solvent molecules can be found in close proximity to the solute, as indicated by the dark blue color. Analyzing the RDFs between the phenoxide and the solvent’s COM (Figure 8(f),(g)), we find a minimum at approximately 4.0 Å. Integration reveals to a coordinating number of three solvent molecules. The same procedure reveals a coordination number of roughly eight molecules by integration up to 6.5 Å for the N-terminus, Figure 8(g). However, the most strongly interacting solvent molecules all cluster around the phenoxide terminus forming hydrogen bonds. Hence, we picked three solvents, see Figure 8(h), for the microsolvated cluster. Interestingly, chloroform with a permittivity constant of ϵ = 4.90 is significantly less polar than methanol but shows strong and directed interactions with the solute. Calculation of the interaction energy of the DFT-optimized cluster yielded −17.9 kcal mol^–1^.

In contrast to methanol and chloroform, the resulting bar plot of BM in DMSO with a dielectric constant of ϵ = 46.7 (Figure 9(a)) shows a steady decrease in Free Energy until it eventually converges toward bulk starting from the solvent ID 31. Simultaneously, the Free Energy difference between the most strongly interacting solvent and bulk is only 2.5 kcal mol^–1^, indicating a weaker solute-solvent interaction than methanol or chloroform.

**Figure 9:**
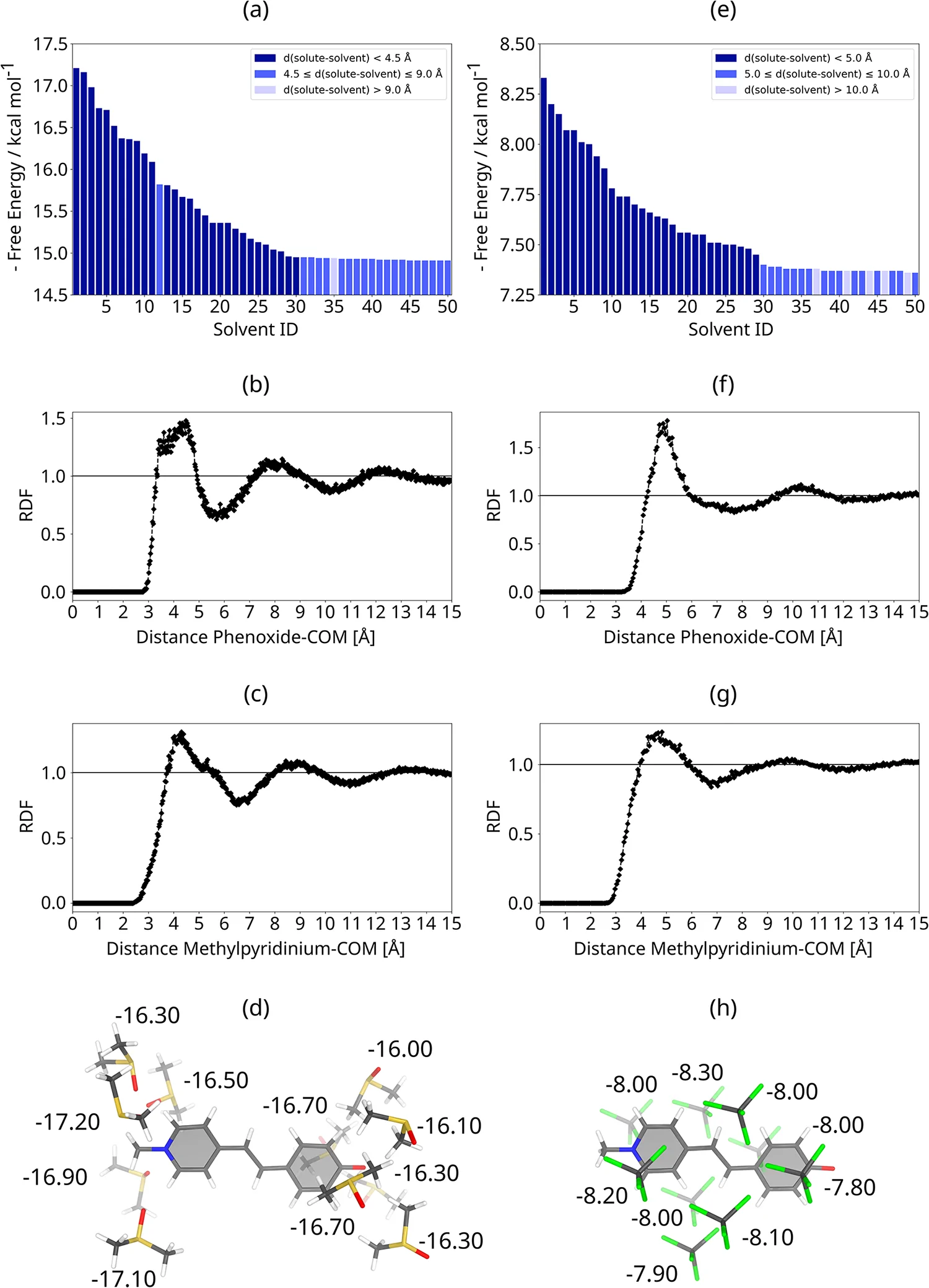
Microsolvation of Brooker’s merocyanine dye in DMSO and tetrachloromethane. Bar plots resulting from FEBISS 2 workflow, showing solvent molecules ranked according to their interaction strength with user-defined solute-solvent distance thresholds, and RDFs around the phenoxide moiety and the methylpyridinium group, are given for DMSO (a–c) and for tetrachloromethane in (e–g), respectively. The BM-(DMSO)_11_ and BM-(CCl_4_)_9_ microsolvated structures are given in (d, h). Free energies of placed solvent are given in kcal mol^–1^.

The first 30 solvent molecules, collectively form the first solvation shell in a relatively uniform manner.

The solvation sites can be roughly divided into four regions (see Figure S06): the strongest interactions occur at the methylpyridinium moiety in a radial arrangement, followed by slightly weaker interactions around the phenoxide group, and medium to weak interactions in the apolar region of the extended π-system as well as at the methyl group. Based on this spatial distribution, selecting the first 11 solvent molecules appears reasonable, further supported by the energy gap between solvent ID 11 and solvent ID 12 in the bar plot. This decision is mostly corroborated by the evaluation of the integrated RDFs, Figure 9(b),(c). In the case of the phenoxide moiety, integration from 0.0 to 5.5 Å yielded six solvent molecules in the first coordination sphere, while in the case of methylpyridinium integration from 0.0 to 5.0 Å, resulted in four solvent molecules. The resulting microsolvated structure with 11 solvent molecules BM-(DMSO)_11_ is given in Figure 9(d).

As an example of an apolar solvent, we investigated BM in tetrachloromethane (ϵ = 2.24). From the bar plot depicted in Figure 9(e), one again can see that there is a steady decline in interaction energy with 29 solvent molecules being in close proximity to the solute. However, the differences in Free Energy between the strongest interacting solvent and the bulk value is only 1 kcal mol^–1^, indicating very weak overall solvent-solute interactions.

A more pronounced energy gap was found between solvent ID 9 and solvent ID 10. Unlike the previous examples, however, the first nine solvent molecules are located around the apolar phenyl units of the solute. This finding is in agreement with chemical intuition but it makes the RDFs around the polar moieties in Figure 9(f),(g), included here for completeness, superfluous. The microsolvated cluster BM-(CCl_4_)_9_ is depicted in Figure 9(h).

The robustness of the methodology was further assessed for Brooker’s dye in DMSO by comparing the default center-of-mass (COM) placement against placements using the sulfur or oxygen atom as the reference center (see Supporting Information Figure S05). While these choices lead to moderate differences in both the interaction strength profiles and the placement of individual solvent molecules, the conclusions remain unaffected by the choice of reference center, and all choices yield plausible results.

### A Homogeneous Catalyst for Olefin Metathesis

In a previous study, we investigated olefin metathesis mediated by a Schrock-type molybdenum catalyst,^84–86^ highlighting how conformational flexibility of the catalyst governs the reaction pathway and is impacted by explicit environments.^71,87^ Building on these insights, we now investigate how microsolvation affects the molecular properties. As a test example, we chose Mo­(N-2,6-Me_2_C_6_H_3_)­(CHCMe_3_)­(MesH_2_)­(OTf)_2_ (1, R = Mes = 2,4,6-(CH_3_)_3_C_6_H_2_, OTf = CF_3_SO_3_),^85,86^ abbreviated as MoCat, in its catalytically most active *anti*-isoform and studied its interaction with dicholoromethane (DCM).

Applying the FEBISS 2 workflow, we obtain a bar plot (see Figure S08), from which we chose 11 DCM molecules, resulting in MoCat-(DCM)_11_. Overall, the energy difference between the most strongly interacting DCM molecule and the bulk value is ca. 2.5 kcal mol^–1^ and starting from solvent ID 12, a smooth convergence towards the bulk value Δ*E*_vv_ of −6.0 kcal mol^–1^ is visible. As there are many weakly interacting solvent molecules in close proximity to the solute, this finding suggests that extended regions of the solute surface do not show highly favorable solute-solvent interactions. Indeed, the DCM molecules of the resulting MoCat-(DCM)_11_ cluster, (Figure 10(b)), are centered around the triflate ligands, with the solvents exhibiting the lowest Free Energy values being oriented toward the sterically unhindered, terminal oxygen atoms.

**Figure 10:**
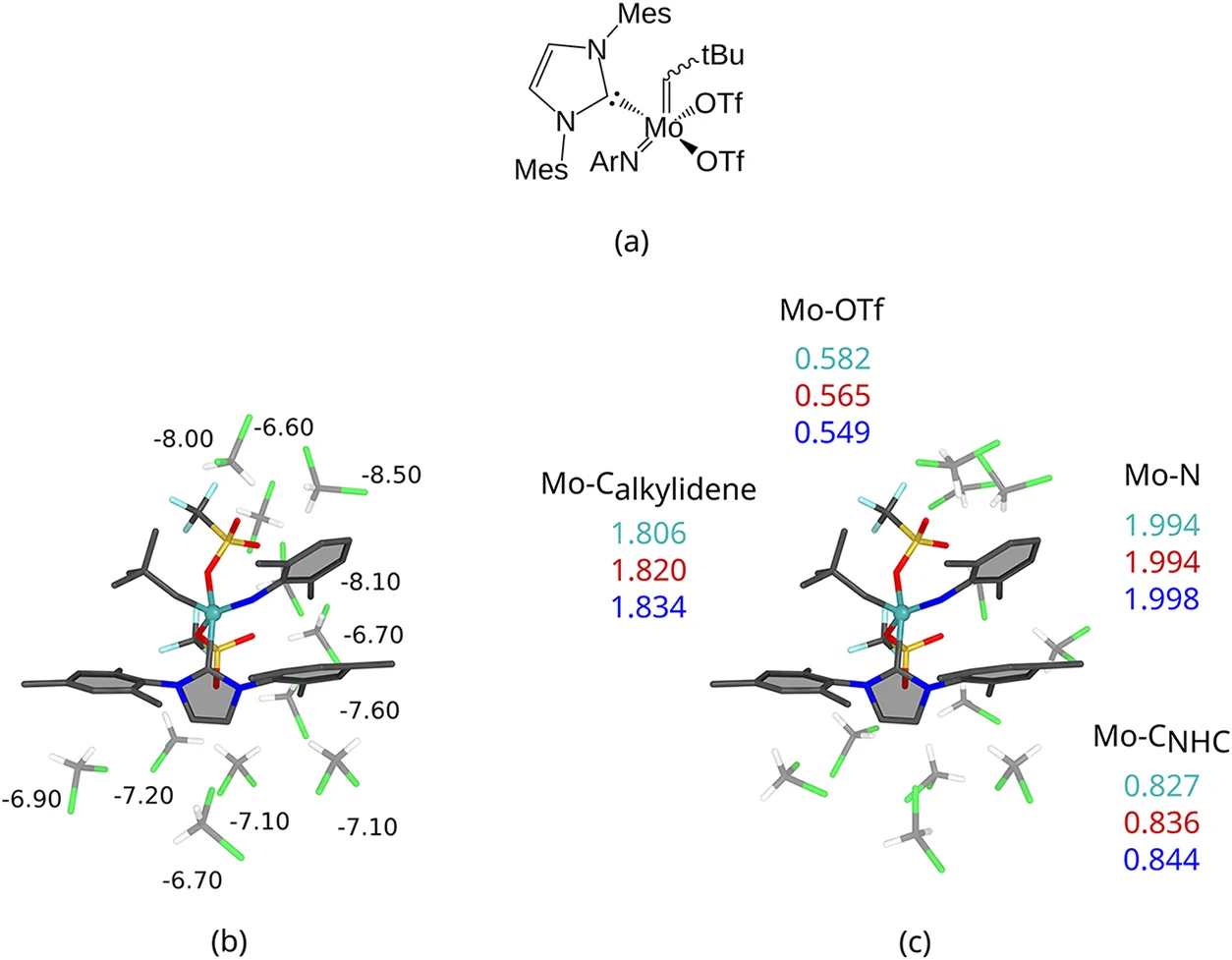
(a) Lewis-structure of the molybdenum imido alkylidene NHC catalyst. (b) Microsolvated MoCat-(DCM)_11_ as obtained from FEBISS with the placed solvent Free Energies given in kcal mol^–1^, (c) and the QM/MM optimized structure of MoCat-(DCM)_11_. Bond order indices are calculated as DFT single point on MoCat-(DCM)_11_. Implicit solvent and gas phase values were obtained as single point calculations on the MoCat geometry after removing the solvent molecules. Hydrogen atoms are omitted for clarity. Depicted values are Mayer bond order indices for the gas phase calculation (cyan), implicit solvent calculation (orange), as well as the microsolvation calculation (blue).

Subsequently, the MoCat-(DCM)_11_ cluster was optimized using QM/MM (see Post-Processing in the Computational Methodology for details). The resulting complex is shown in Figure 10(c).

To assess the influence of solvation models on the electronic structure, we performed a DFT single point calculation with additional C–CPM on the MoCat-(DCM)_11_ complex.

As a proxy for reactivity, we analysed Mayer bond order indices on (1) the Mo–C_alkylidene_, (2) the Mo-OTf, (3) the Mo–N-, and (4) the Mo–C_NHC_-bond for the three models. The results of the calculations in the gas phase, in implicit solvent, and using the microsolvated approach are given top-down in Figure 10(c). Bond orders for the Mo–C_alkylidene_, Mo–N, and Mo–C_NHC_ centers increase progressively from the gas phase to implicit solvent and further under microsolvation. In contrast, the Mo-OTf bond order exhibits a downward trend. While the magnitude of these shifts is bond-specific, the transition from implicit solvent to microsolvation induces changes comparable in scale to those observed when moving from the gas phase to a continuum model. This finding clearly indicates the impact of explicit solvation on the electronic structure and thus on reactivity.

## Discussion

Our newly devised FEBISS 2 algorithm allows for the generation of solute-solvent clusters with arbitrary rigid solvents, placing and orienting them based on explicitly quantified solute–solvent interaction strengths rather than geometric or stepwise heuristics. Thereby, it relies on GIST, which was shown to give reliable insights into solvation thermodynamics of molecules over a range of different solvents and molecular species. While all investigated examples are neutral, charged systems can also be treated. Despite the broad applicability of our methodology, we would like to note two points to be kept in mind: (i) Our algorithm is based on classical MD simulations. As such, the reliability of the results are dependent on the choice of the force field parameters and their accuracies. For ambient temperature and pressure, the Generalized Amber Force Field in conjunction with RESP partial charges give good results for a large number of different species.^66^ However, a GIST analysis can be applied to any force fields as long as the trajectory, as well as parameter and topology files can be read into CPPTRAJ. Especially, machine-learning potentials could give promising and accurate results at low computational costs but would demand adaptation of the GIST evaluation because typically topologies are not explicitly stored. (ii) GIST requires the solute to remain rigid throughout the simulation. For molecules with a high degree of conformational flexibility, it is therefore necessary to perform a conformer search prior to the actual GIST runs in order to identify the most relevant configurations. Each of which can then be subjected to FEBISS treatment.

It is important to note here that we assign Δ*G*_solv_ values that characterize the interaction strength between a solvent molecule and the solute at a specific position in space. These values are based on classical MD simulations. Unlike geometric extraction criteria used in other top-down approaches, this provides a direct, physically grounded quantification of the solvent interaction environment rather than an a posteriori assessment of user-selected clusters. With our approach, we do not primarily aim to obtain a converged solvation Free Energy Δ*G*_solvation_ of our solute. While this quantity could be obtained from our data, our placed solvent molecules are located in positions that are averages over the entire trajectory and do not necessarily correspond to local energy minima. To study Δ*G*_solvation_ would require additional conformational sampling to identify the lowest energy conformer or ensemble of conformers. However, obtaining converged Δ*G*_solvation_ from microsolvation approaches remains challenging despite recent progress.^88^ The purpose of our methodology is to identify a small set of solvent molecules that strongly interact with the solute, that modify its electronic structure and, consequently, its reactivity, primarily for mechanistic investigations.

The performance of the FEBISS 2 approach is well illustrated by the presented examples. In the case of urea, the obtained results are in good agreement with available ab initio molecular dynamics data,^79^ 3D-RISM calculations,^35^ and other microsolvation approaches.^37^ A quantitative, method-to-method comparison against these approaches offers only limited additional insight, because the underlying placement algorithms differ fundamentally in how solvent molecules are selected and assigned; the qualitative agreement observed here nevertheless supports the physical plausibility of the FEBISS 2 results. Importantly, differences relative to our initial FEBISS implementation, where an elemental density-based solvent placement was used rather than the quaternion averaged placement of our FEBISS 2 methodology, are small (see SI for a detailed discussion). These results support the robustness of the methodology and confirm that the new orientation procedure preserves the plausibility of our original approach while extending it to arbitrary rigid solvents. A key factor contributing to this robustness is the voxel-based placement scheme, where voxel sizes are smaller than a single solvent molecule. Solvent placement is achieved by the identification of voxels of high occupancy. In most cases, several adjacent voxels are needed to account for one solvent molecule. The subsequent depletion of occupancy of these voxels generally prevents solvent overlap.

To further corroborate the robustness of FEBISS 2, we compared, for Brooker’s dye in DMSO, the default center-of-mass (COM) placement against placement using the sulfur or oxygen atom as the central point. Although the choice of the central point has a moderate effect on interaction strength and individual solvent placement, it does not alter the overall conclusions. We nevertheless consider COM placement the most generally applicable and unbiased default, as it provides a consistent, solvent-independent definition across chemically diverse solvents. While selecting a chemically specific atom, such as the oxygen atom in hydrogen bonding solvents, may improve the representation of certain local interactions in individual cases, no single atomic reference is universally appropriate.

Across different systems, FEBISS 2 also captures clear variations in solute–solvent interaction strengths a level of quantitative resolution not offered by geometry- or shape-based extraction methods. For instance, comparing DMSO solvation of (+)-borneol and Brooker’s merocyanine dye shows that interaction strengths are highly system dependent: the strongest DMSO interaction in (+)-borneol is approximately 4.5 kcal mol^–1^ stronger than bulk, whereas for Brooker’s merocyanine dye it is only about 2.5 kcal mol^–1^. A comparison of interaction strengths across four solvents for Brooker’s merocyanine dye further indicates that solute-solvent interaction strength cannot be directly inferred from solvent polarity. For example, chloroform with a dielectric constant of 4.9 shows pronounced directed interactions with the solute indicated by an interaction strength of 3.3 kcal mol^–1^ compared to bulk, wheres the more polar DMSO only shows a maximum interaction of 2.5 kcal mol^–1^. The strong interaction of chloroform is further corroborated by analysis of the interaction strength in comparison to methanol, which is known for its pronounced hydrogen bonds. For chloroform, we find an interaction strength of −17.9 kcal mol^–1^ vs −23.3 kcal mol^–1^ for methanol from DFT analysis, indicating a strong solute-solvent interaction despite a low polarity.

In the case of MoCat, microsolvation was shown to have a profound impact on the electronic structure, as indicated by changes in the Mayer bond order as a proxy. Although this system is generally considered to be only weakly affected by solvent, the observed changes are comparable in magnitude to those arising from moving from the gas phase to implicit solvation, which is widely regarded as important in computational modeling.

Hence, with our methodology, we not only understand how local solvation influences the electronic structure of the complex but more broadly, we can now study how explicit solvent coordination can reshape catalytic landscapes beyond what is accessible through continuum descriptions alone, providing a general route to incorporating microsolvation into reactivity studies of organic and organometallic compounds.

## Conclusion

Identifying, ranking, and placing the small number of solvent molecules that most significantly impact a solute’s molecular properties and reactivity remains a central and largely unsolved problem for microsolvated cluster–continuum models: existing strategies either build up solvation shells stepwise, introducing an order dependence and a bias toward gas phase-like geometries, or extract clusters from condensed-phase trajectories based on user-defined geometric criteria, without directly quantifying solute–solvent interaction strength.

We have developed FEBISS 2, a computational methodology based on classical MD simulations and Grid Inhomogeneous Solvation Theory addressing this problem in a general and physically motivated way. Starting from a full condensed-phase MD simulation, our approach identifies the most strongly interacting solvent molecules based on thermodynamic quantities and rigorously places and orients them around the solute using a statistically averaged, order-independent orientation procedure. Therefore, our methodology does not require user-defined geometric selections criteria and avoids artifacts due to stepwise optimization.

Through the introduction of a new, quaternion-based orientation procedure together with the possibility to use a center-of-mass-based solvent substructure, our protocol is now fit to study any rigid solvent, including a broad range of commonly used organic solvents relevant to homogeneous catalysis and reaction mechanism exploration.

FEBISS 2 is applicable across a number of different systems as long as the underlying force field is able to capture the interactions; a curated set of ten commonly used, pre-parameterized solvents further enables immediate, out-of-the-box use.

For the Mo imido alkylidene NHC catalyst used in olefin metathesis, the impact of explicit microsolvation on the electronic structure was comparable in magnitude to that of moving from the gas phase to an implicit solvent description. In a broader context, these observations highlight that even systems commonly assumed to be only weakly affected by their environment can exhibit significant solvent-induced electronic effects.

By enabling the automated generation of physically meaningful microsolvated models, FEBISS 2 provides a practical route to incorporating explicit solvation into quantum chemical investigations of molecular structure, electronic properties, and reactivity. This is particularly relevant for exploration of reaction mechanisms, where many different configurations and conformations need to be evaluated, and in transition-metal catalysts, where evaluation of the electronic structure requires expensive wave function methods and full condensed-phase QM treatments remain computationally prohibitive. FEBISS 2 is therefore particularly suited to cases where condensed-phase conformations of the solute are important, where an explicit, quantitative ranking of solute–solvent interaction strength is required rather than a geometrically defined cluster, or where the solvent of interest is *not* water, that is, solvents, for which existing density-based placement approaches are generally not applicable.

Incorporating microsolvation therefore represents an important step toward bridging the gap between idealized computational models and real chemical conditions, ultimately improving the predictive power of theoretical studies in catalysis and reactivity.^89^

## Supplementary Material



## Data Availability

All Cartesian coordinate files of the optimized structures subjected to the GIST simulations, and resulting microsolvated clusters shown here are available on Zenodo under doi.org/10.5281/zenodo.20275360. Our code is available via https://github.com/podewitzlab/febiss and can be used free of charge.

## Notes

Implementation details of FEBISS 2 and its usage; comparison of FEBISS and FEBISS 2; details on the determination of solvent orientations and symmetry correction; additional information on Brooker’s dye and the molybdenum catalyst and a list of predefined solvents that are supported in FEBISS 2 out-of-the-box as well as force field parameters of all solutes (PDF)
