# Supplementary material for: Quantum Chemical Microsolvation via Free Energy–Based Solvation Site Identification
Source: J Chem Theory Comput. 2026 Sep 8;22(18):9640–54. doi: 10.1021/acs.jctc.6c00994 (PMC13602899; doi:10.1021/acs.jctc.6c00994)
Supplement: Supplementary file 1 [file ct6c00994_si_001.pdf]

Quantum Chemical Microsolvation via Free Energy–Based Solvation Site Identification Supporting Information

Lukas B. Magenheim and Maren Podewitz*

Institute of Materials Chemistry, TU Wien, Getreidemarkt 9, 1060 Vienna, Austria

E-mail: maren.podewitz@tuwien.ac.at

1 Implementation of FEBISS and Usage

The FEBISS package was developed in Python3 and can either be used through a command line interface (CLI) or through a graphical user interface (GUI). It is available free of charge on Github.[1] Upon installation, the user has access to three different subprograms accessible via CLI, *febiss_setup*, *febiss_settings*, and *febiss*.

febiss_setup eases the installation of the required packages, and downloads and installs the appropriate CPPTRAJ version. Per default, febiss_setup tries to compile CPPTRAJ with openMPI and CUDA support.

febiss_settings facilitates the setup of the actual FEBISS analysis via an interactive shell. At the end of the CLI dialog, an easy-to-access and editable YAML file is created which contains all settings for the FEBISS run. These include: i) the path to the simulation trajectory and parameter/topology file, ii) information on the solvent used in the simulation, iii) settings for the subsequent GIST analysis, iv) settings for optional RDF analyses, and v) settings for plotting the FEBISS results in an interactive bar plot. Please consult the

manual on GitHub for an explanation of all possible settings and acceptable values.

febiss finally takes this YAML file as argument and carries out the actual analysis by interfacing to a modified version of CPPTRAJ 6.29.1, as described in the main text. To compute the total number of equivalent orientations of the solvent molecule – necessary for the symmetry cleanup procedure (see below) – a point group analysis is performed using the symmetry module of pymatgen[2,3] package. Finally to compute the mean orientation of the solvent molecules, an implementation for the calculation of the mean quaternion according to Markley et al.[4] was included from Github[5] into the code.

2 Force Field Parameters and Average Box Sizes of the MD Simulations

Force field parameters for all solutes can be found in the Appendix Tables S03 to S16.

Table S01 gives the average size of the simulation boxes of the test systems discussed in the manuscript, i.e., urea in water, (+)-borneol in DMSO, Brooker's merocyanine dye (BM) in methanol (MeOH), chloroform, tetrachloromethane, dimethyl sulfoxide (DMSO), and the molybdenum imido alkylidene N-heterocyclic carbene catalyst (MoCat). This information was extracted via the *avgbox* function in CPPTRAJ[6] using all 10000 frames of the simulation trajectory.

Table S01: Average box sizes of the test systems, obtained with the *avgbox* function in CPPTRAJ using all simulation frames.

System	X [Å]	Y [Å]	Z [Å]
Urea-H_2O	45	45	45
Borneol-DMSO	54	48	46
BM-MeOH	57	62	62
BM-$CHCl_3$	59	63	59
BM-CCl_4	58	57	66
BM-DMSO	54	53	51
MoCat-DCM	43	43	51

3 Comparison of FEBISS and FEBISS 2

For a direct comparison between our original FEBISS implementation and FEBISS 2, we chose the same simulation of urea in water as described in the main text. To match the conditions of our previous algorithm, which was restricted to the placement of water only, we chose the central point P_0 of the water molecule to be the oxygen atom in FEBISS 2. As depicted in the superposition of the resulting urea-$(H_2O)_{10}$ cluster, Figure S01, the oxygen atoms are placed at the exact same locations in both algorithms. The same holds true for coordinates of the oxygen atoms of all the other placed water molecules. This is expected since the underlying simulation is the same and the placement is based on the density distribution of the water molecules around the solute. Differences in the overall conformation of the resulting cluster arise. However, both algorithms adopt different approaches to determine the orientation of the placed solvent molecule: In the original FEBISS implementation, the hydrogens are placed based on the hydrogen density in proximity of the placed oxygen atom assuming a predefined geometry (for a detailed description we refer the reader to the original publication[7]) but the density peak of the 2nd hydrogen atom could not always be uniquely defined. In FEBISS 2, in contrast, an averaged orientation is calculated considering each simulation frame. For this average, all water molecules, which were registered to be inside the respective voxels the oxygen atoms were placed into, are taken into account. This approach, however, is more complicated in the case of symmetrical solvent molecules, because it requires taking into account chemical equivalency of atoms that are distinguishable in the MD simulations, as illustrated in the main manuscript.

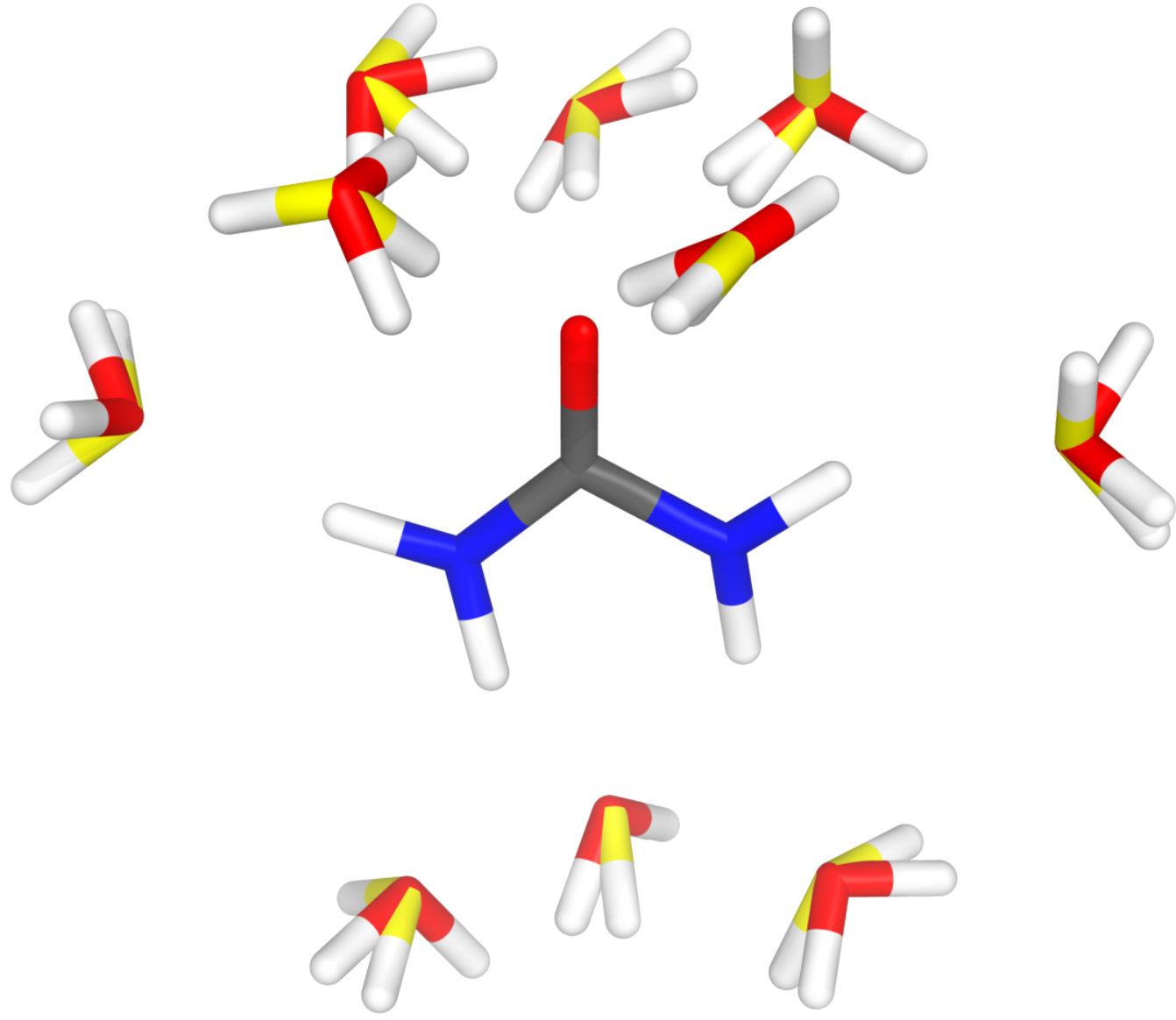

Figure S01: Urea-$(H_2O)_{10}$ cluster resulting from the original FEBISS implementation (oxygen atom in yellow) and the new FEBISS 2 algorithm (oxygen atom in red).

Also, the way of measuring the distance between solvent and solute differs slightly between the implementations: In the original FEBISS version, only heavy (non-hydrogen) atoms and polarized hydrogen atoms of the solute were considered. In FEBISS 2, distances are calculated between all pairs of atoms between the solute and the placed solvent, regardless of their nature and polarity. In conjunction with differently oriented water molecules, this may result in slight differences in the solute-solvent distances, even though the same cutoff values of 3.0 and 6.0 Å were considered, see Figures S02 and S03.

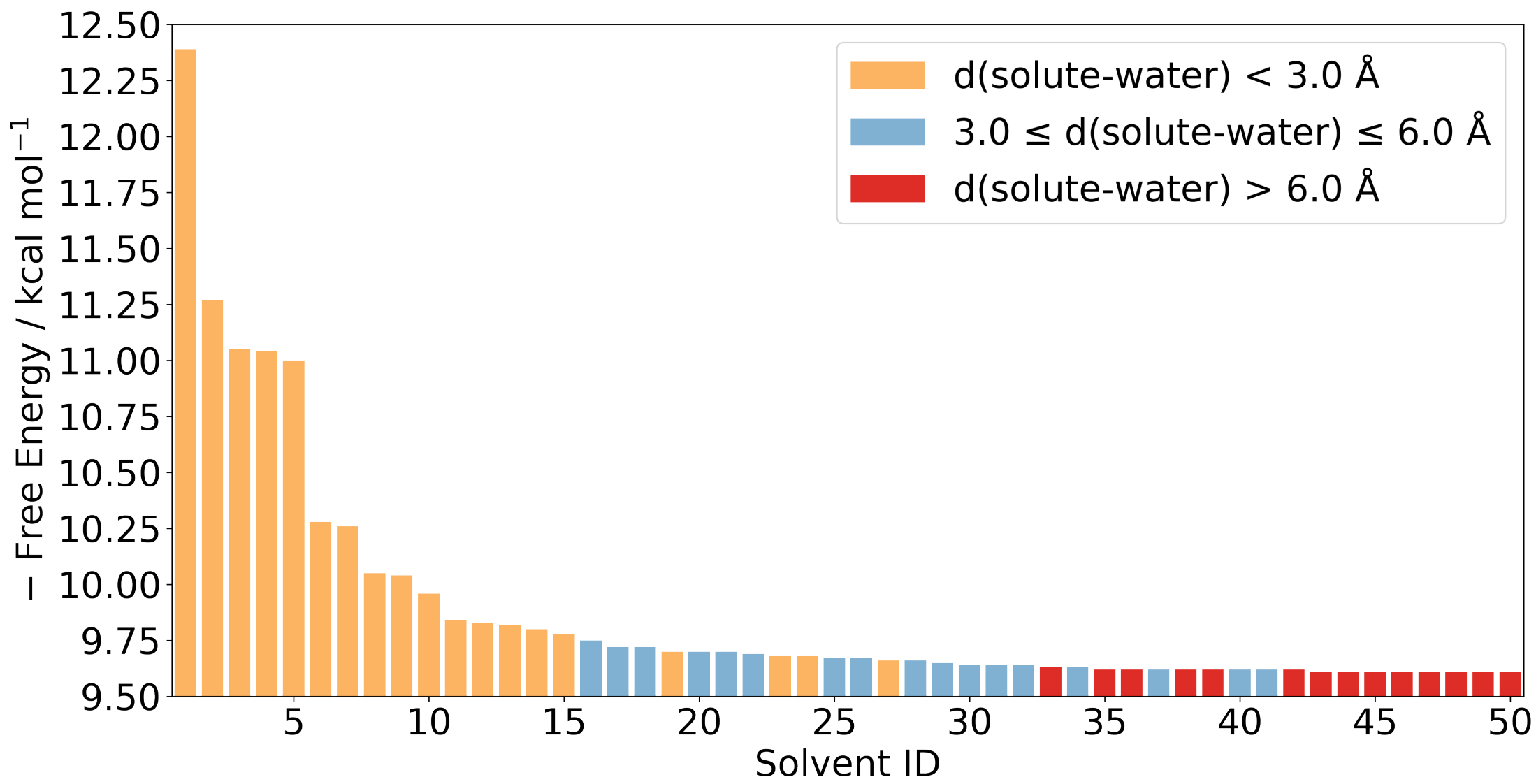

Figure S02: Bar plot obtained from the original FEBISS implementation.[7] The first ten solvent molecules were chosen for the urea-$(H_2O)_{10}$ cluster as depicted in Figure S01 (yellow oxygen).

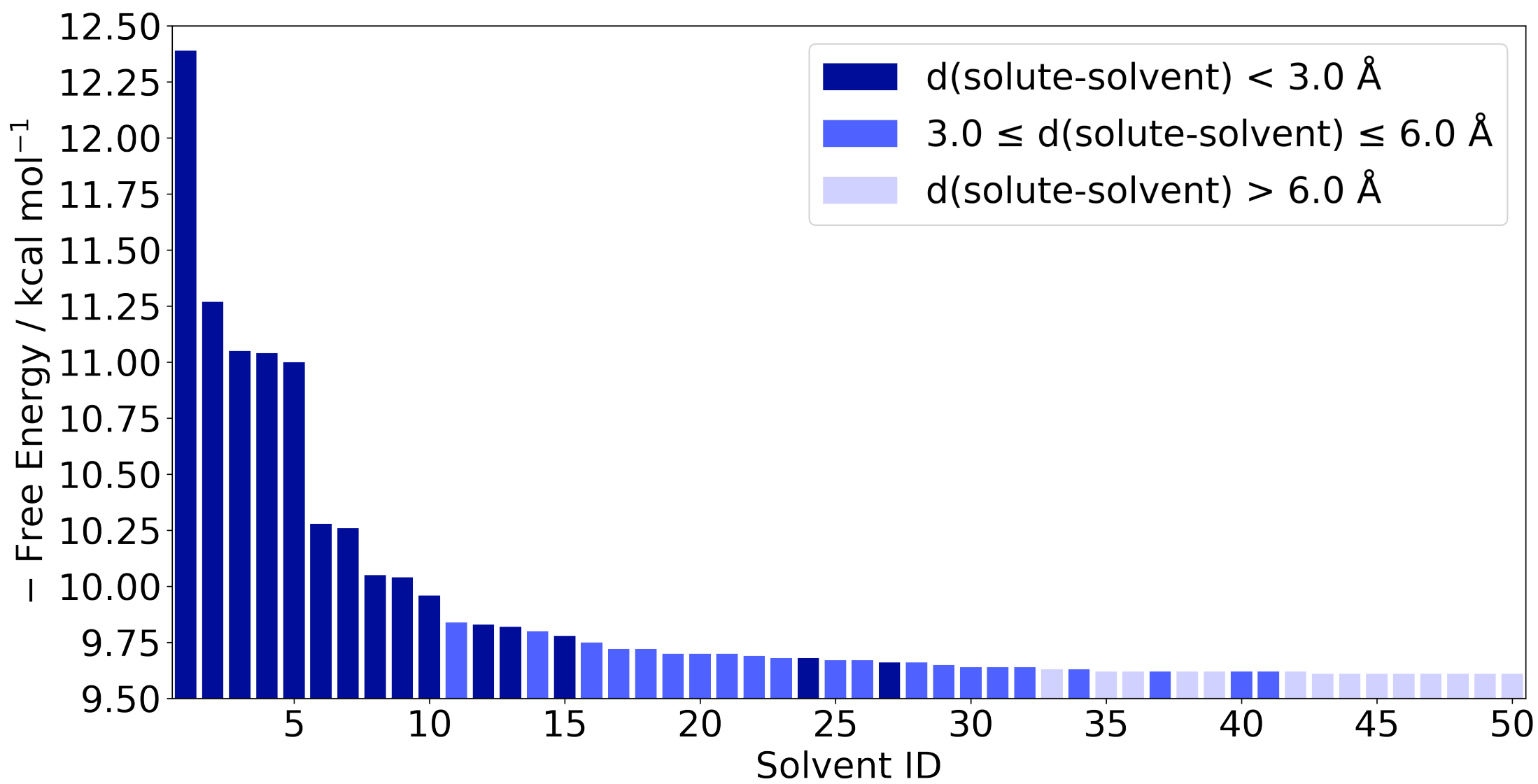

Figure S03: Bar plot obtained from new FEBISS 2 implementation with P_0 set to be the water's oxygen atom. The first ten solvent molecules were chosen for the urea-$(H_2O)_{10}$ cluster as depicted in Figure S01 (red oxygen).

4 Impact of Voxel Size on Solvent Placement and Timing

To assess the sensitivity of the FEBISS 2 placement procedure to the chosen voxel size, additional calculations were performed for urea in water using voxel sizes of 0.25 Å, 0.75 Å, and 1.0 Å, in addition to the default voxel size of 0.5 Å used throughout the main manuscript. The resulting solvent distributions are shown in Figure S04.

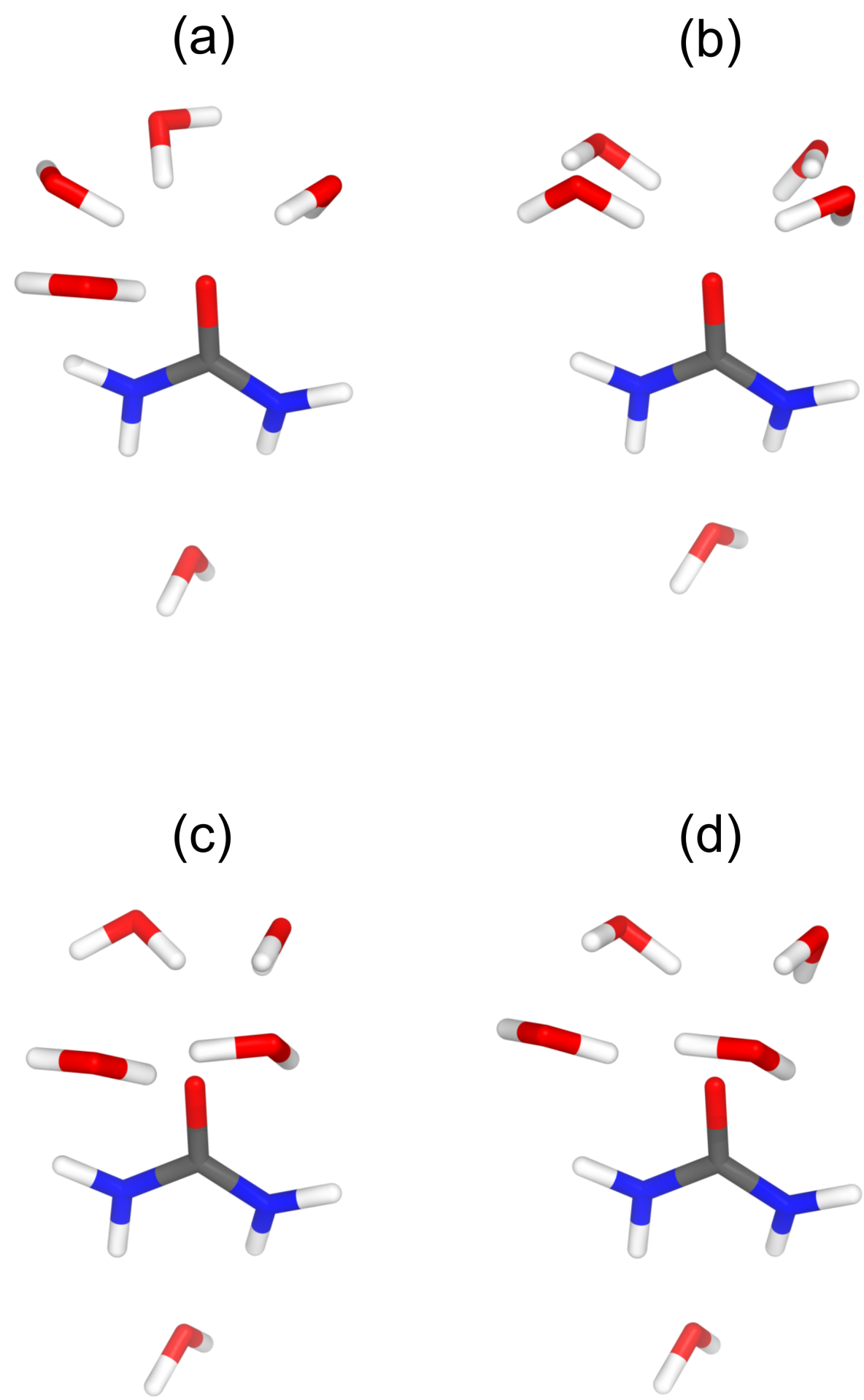

Figure S04: Urea-$(H_2O)_4$ clusters obtained with a voxel size of (a) 0.25 Å, (b) 0.50 Å, (c) 0.75 Å, and (d) 1.00 Å.

While the voxel size has a small, measurable effect on the resulting solvent distribution, the placements obtained across all tested voxel sizes remain remarkably similar. Smaller voxel sizes provide higher spatial resolution but come at increased computational cost. Voxel sizes that are too small, however, lead to low occupation numbers per voxel, resulting in less

robust statistics. Larger voxel sizes reduce computational cost but result in a lower spatial resolution of the solvent placement, leading to a higher likelihood of placement artefacts. A voxel size of 0.5 Å was found to be a good compromise between spatial resolution and computational cost, and was therefore used as the default value throughout this work.

The FEBISS analysis itself is comparatively inexpensive. For the urea-in-water system, the GIST analysis was performed on a single desktop CPU (AMD Ryzen 9 5950X, 16 cores) and required approximately 3 min 4 s for the finest grid spacing (0.25 Å) and 2 min 39 s for the coarser grid spacings (0.5, 0.75, and 1.0 Å). The FEBISS analysis therefore represents only a negligible fraction of the total computational effort, compared to the setup, equilibration, and production MD simulations.

5 Impact of Substructure Determination on Solvent Placement

To assess the robustness of the methodology with respect to the choice of the central atom, solvent placement for Brooker's dye in DMSO was additionally performed using the sulfur and oxygen atoms as reference centers, rather than the default center-of-mass (COM) placement. These choices lead to differences in both the bar plots and the placement of individual solvent molecules, see Figure S05 (a to c). When the oxygen atom is used as the reference center Figure S05 (c), the resulting solvent distribution is more localized indicated by stronger interaction with the solute, which is visible in the corresponding bar plots. However, in all three cases, the most strongly interacting solvents are located around the N-terminus of Brooker's merocyanine dye. This robustness is further corroborated by the (+)-borneol case, where, despite using COM placement rather than explicitly placing the oxygen atom, the correct oxygen position and hydrogen bond to the solute were still predicted (see main manuscript).

(a)

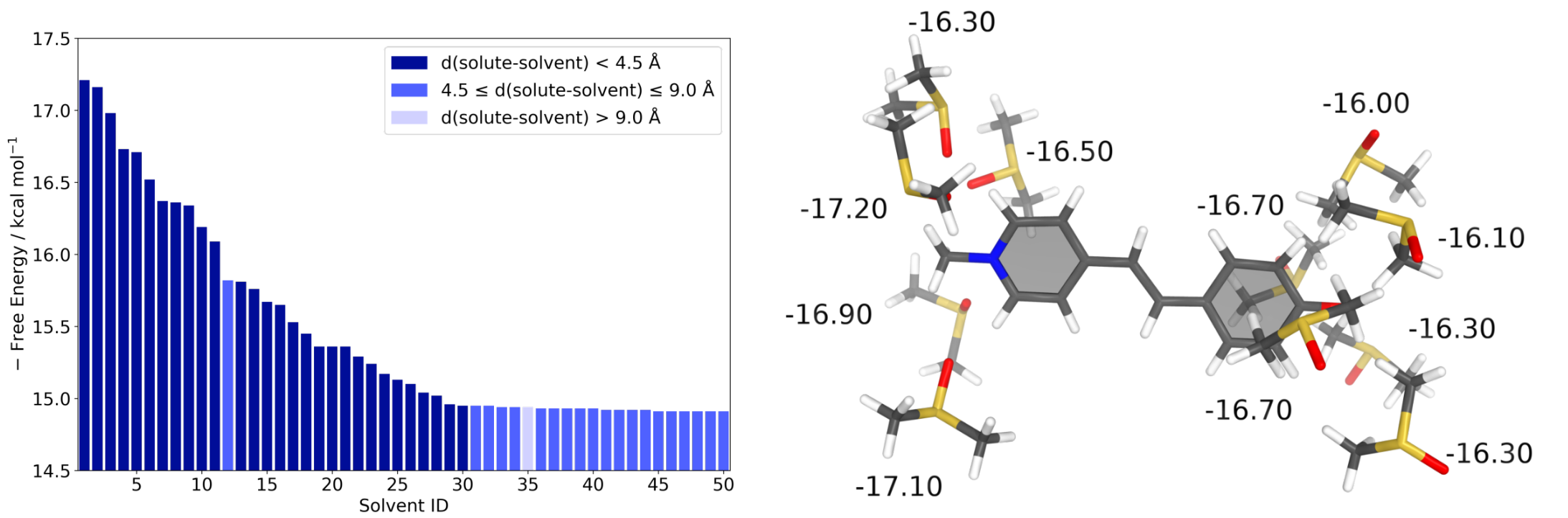

(b)

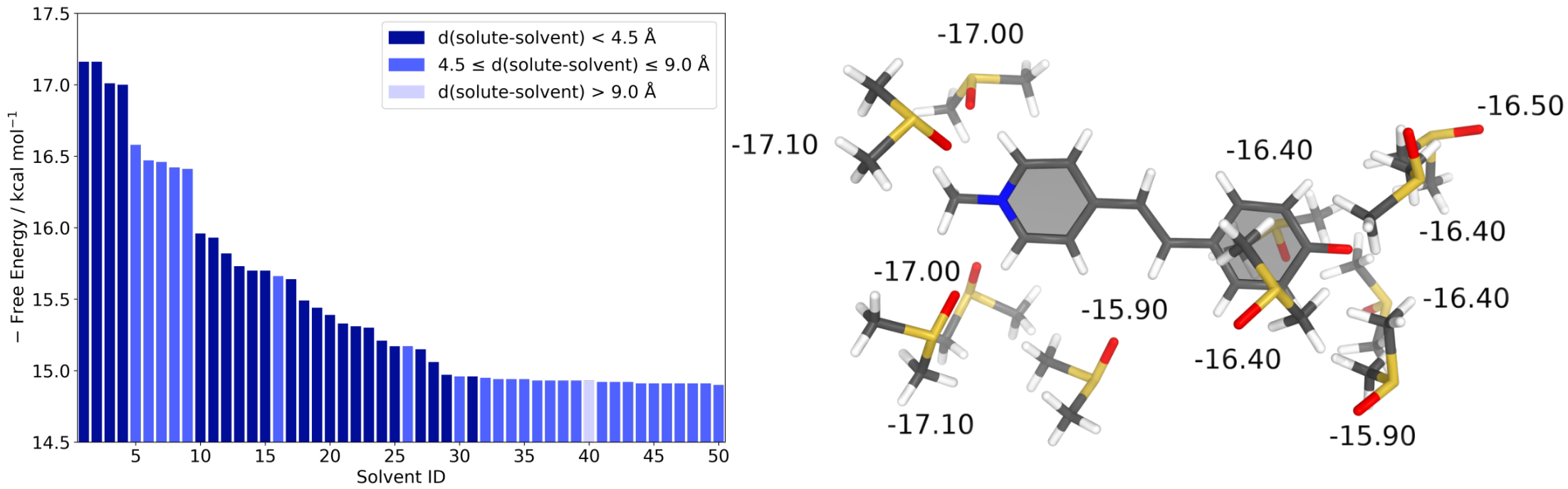

(c)

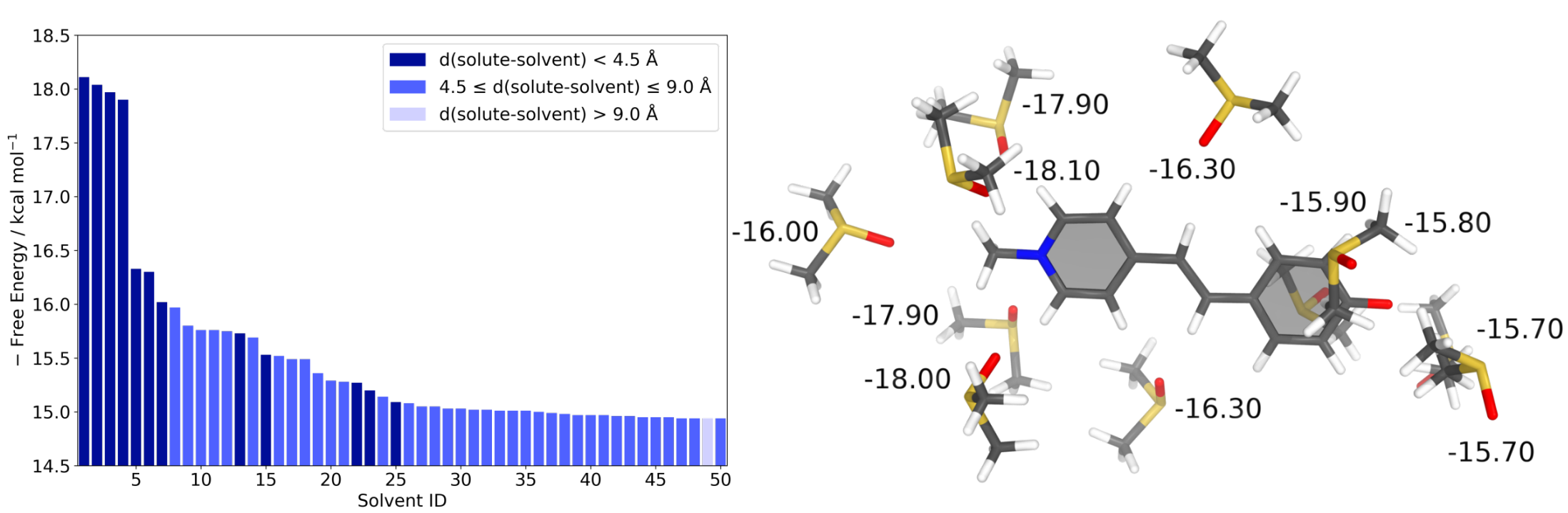

Figure S05: Brooker's dye bar plots and resulting microsolvated in DMSO clusters BM-$(DMSO)_{11}$ obtained using (a) the COM, (b) the sulfur atom, and (c) the oxygen atom as central point P_0.

Despite the dependence of the solvent placement on the choice of the central atom, we recommend the COM placement to yield the most consistent results across all solvents, while also minimizing additional user choices.

6 Determination of Orientation and Symmetry Correction

To determine the orientation of the solvent molecule that was placed in voxel k, our algorithm accesses orientational data stored for every solvent molecule registered in this particular voxel across all simulation frames. These orientations are given as quaternions $\mathrm{q_i}$ which are determined during the calculation of $\mathrm{S_{uv}^{or}}$ of GIST:[8] For each of the predefined solvent molecules, we define three points in the solvent P_0, P_1, P_2 which form a non-zero angle $\angle \mathrm{P_1} - P_0 - P_2$ smaller than 180°. P_0 is a central point in the molecule which can either be the center of mass (COM) or a central atom. P_1 and P_2 are the coordinates of two atoms (see Table S02 for an overview of the predefined solvent molecules). The xy-plane is spanned by these three points with the x-axis coinciding with the vector $\overline{\boldsymbol{P_0P_1}}$. Together with the z-axis perpendicular to this plane, a (solvent) cartesian coordinate system is defined that can be related to the laboratory reference coordinate system by rotation. Here we describe this rotation as a unit quaternion of format $[\mathrm{q_0}, \mathbf{q}]$ and use it to define the orientation of the solvent molecule.

However, since atoms are numbered and, thus, distinguishable in MD simulations, symmetrically equivalent orientations give rise to different quaternions. Taking chloroform as an example which has the point group $\mathrm{C_{3v}}$, the same molecule orientation can be described by three different quaternions, depending on which chlorine atom has been chosen for setting up the solvent coordinate system. Although this fact is not important for calculating $\mathrm{S_{uv}^{or}}$ given a sufficiently long trajectory,[8] it impacts the calculation of an average orientation.

Thus, our program has to correct for this fact: First, the algorithm loads the coordinates

of the solvent molecule used in the MD simulation with a predefined orientation with respect to the lab coordinate system. This solvent molecule acts as reference molecule denoted by the subscript "ref". In the next step, FEBISS 2 calculates all symmetrically equivalent structures of the reference molecule, and stores a) the rotational transformation operations as quaternions $q_a, q_b, ...,$ — in total N rotations yielding equivalent structures — and b) the orientations of the resulting equivalent structures with respect to the lab coordinate system as quaternions $q_1, q_2, ...$ — in total M. Although M equals N, we chose a different letter indices to emphasize the difference between quaternions defining *rotations* between symmetrically equivalent structures (letters) and quaternions defining *orientations* with respect to the lab coordinate system (numbers).

Considering chloroform as an example, we obtain three solvent structures that are equivalent to each other. Their orientation with respect to the lab coordinate system is described by their quaternions q_{ref}, q_1, and q_2. The three quaternions q_a, q_b and q_c describe the rotations $q_1 \rightarrow q_{ref}$ and $q_2 \rightarrow q_{ref}$, and the identity operation $q_{ref} \rightarrow q_{ref}$. Note that we do not need to take mirror or inversion operations into account since these do not occur in classical MD simulations.

Next, FEBISS 2 calculates the distance $\Delta\omega_i$ for each of the quaternions q_i stored for voxel k to q_{ref}, q_1, and q_2 via

$$\Delta\omega_i = 2\cos^{-1}(|q_i \cdot q_M|)$$

where $q_i \cdot q_M$ denotes the dot product between the orientational quaternion of chloroform i and q_M which, here, is q_{ref}, q_1, or q_2.

If q_i is closest to q_{ref} no correction has to be made. If, however, q_i is closest to, say, q_1, then q_i gets transformed into $q_i{}'$ via

$$q_i{}' = q_i \star q_{ref}^{-1} \star q_a \star q_{ref}$$

q_i' is now symmetrically equivalent to q_i but now it is closest to q_{ref}. A similar procedure was described by Hu et al.[9] It is important to note, that for this cleanup procedure to be correct, the central point has to be chosen to stay invariant during the symmetry operations. The center of mass is thus the most robust choice for P_0. The average orientation $\bar{\mathbf{q}}$ of the solvent molecule was calculated according to the maximization procedure given by Markley et al.:[4]

$$\bar{\mathbf{q}} = \arg\max_{\mathbf{q}\in\mathbb{S}^3} \mathbf{q}^T M \mathbf{q}$$

where M is the 4x4 matrix

$$M \triangleq \sum_{i=1}^{n} \mathbf{q}_i \mathbf{q}_i^T$$

.

7 Brooker's Merocyanine Dye

The first 30 dimethyl sulfoxide (DMSO) molecules envelop the Brooker's merocyanine dye in a fairly uniform manner. Figure S06 shows the positions of the sulfur atoms of the placed DMSO molecules which are colored and ranked according to the Free Energy value assigned. Apolar and polar parts of the solute can be easily distinguished and most strongly interacting solvents are located close to the N-terminus.

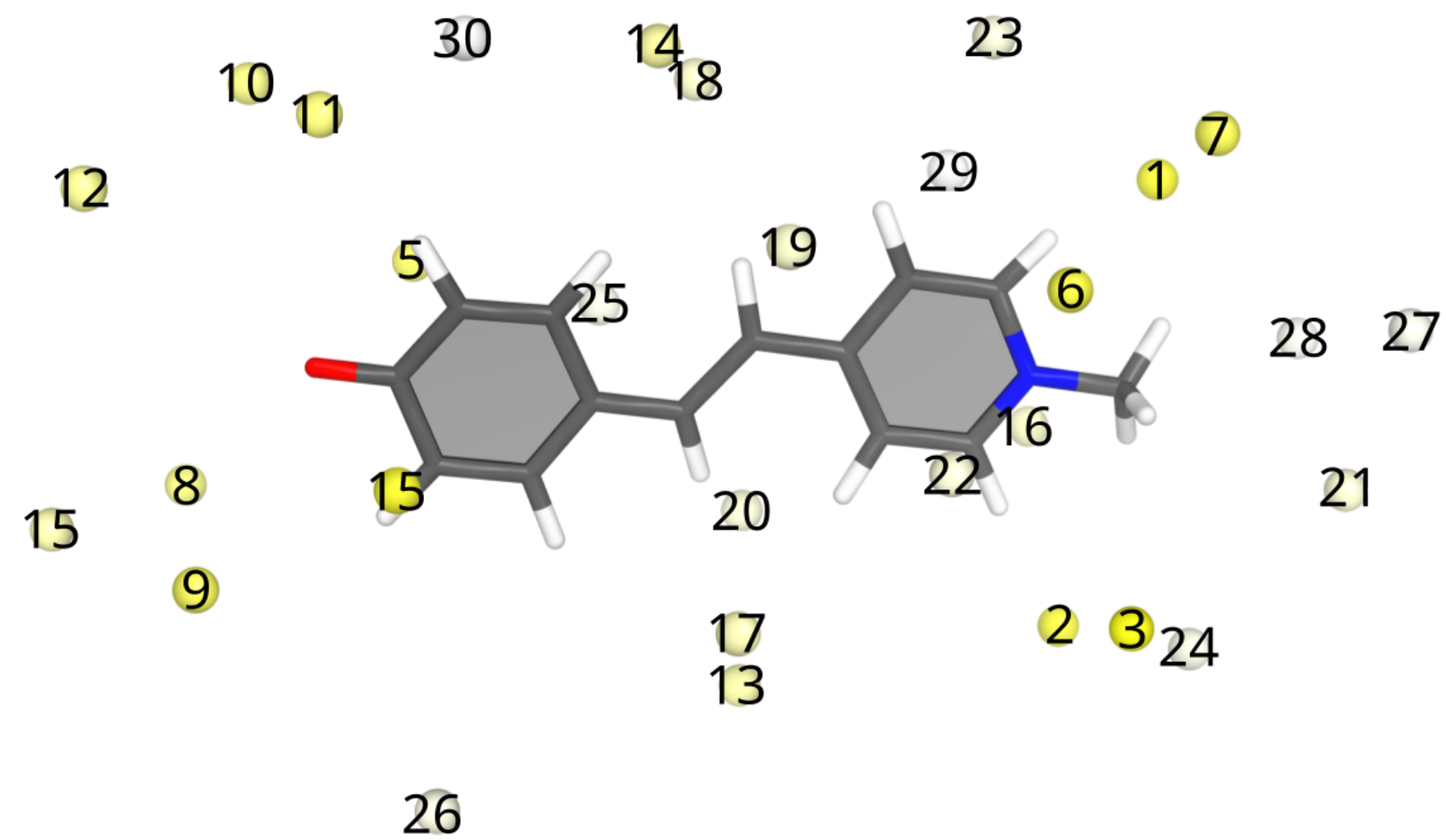

Figure S06: Brooker's dye microsolvated in DMSO ID 1 - 30, without resolving the orientation yet. Only sulfurs are shown for clarity. The shade of the color indicates the corresponding free energy value, the darker the more favorable the interaction, and thus the lower the free energy value.

In the case of methanol as solvent, the first five strongly interacting solvent molecules all cluster around the negatively charged moiety of Brooker's dye (see Figure S07). For the final microsolvated structure, shown in the main manuscript, only the first three solvents were chosen after inspecting the RDF.

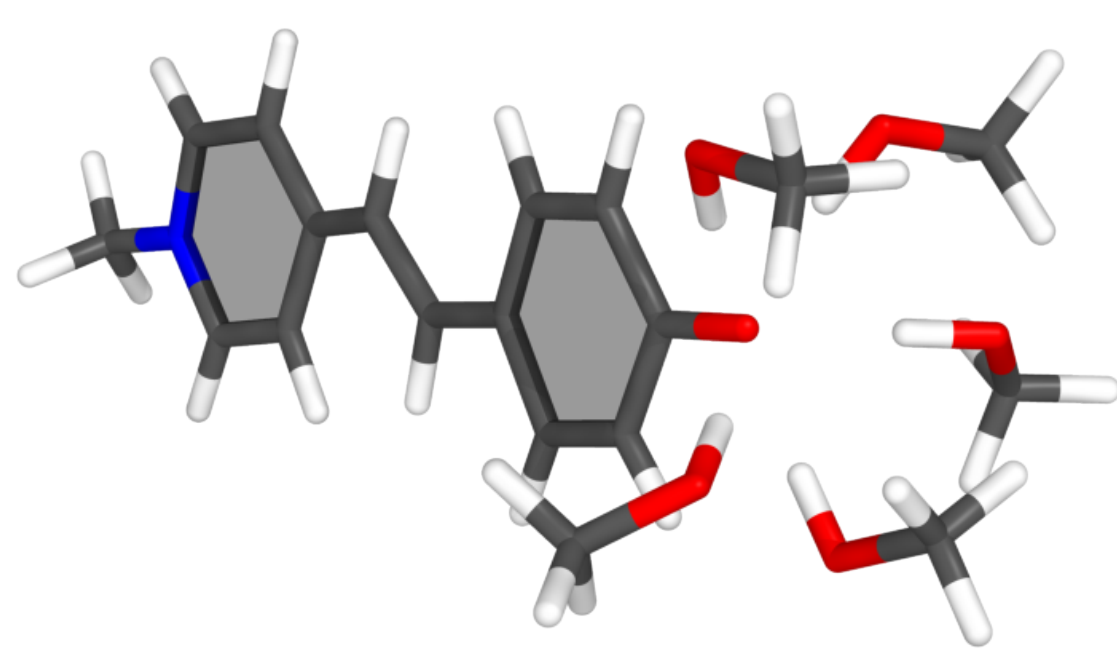

Figure S07: Brooker's dye microsolvated in methanol ID 1 - 5, BM-$(DMSO)_5$. The methanol molecules all group around the phenoxide moiety and form hydrogen bonds.

8 A Homogeneous Catalyst for Olefin Metathesis

Figure S08 depicts the resulting bar plot of the Schrock-type molybdenum catalyst for olefin metathesis (MoCat) in dichloromethane (DCM), obtained from applying the FEBISS 2 workflow (MD simulation, GIST analysis, solvent placement and free energy assignment, ranking, orientation). The color coding indicates the atom-pair wise closest solute-solvent distances, whereas the thresholds were user-defined based on the size of DCM.

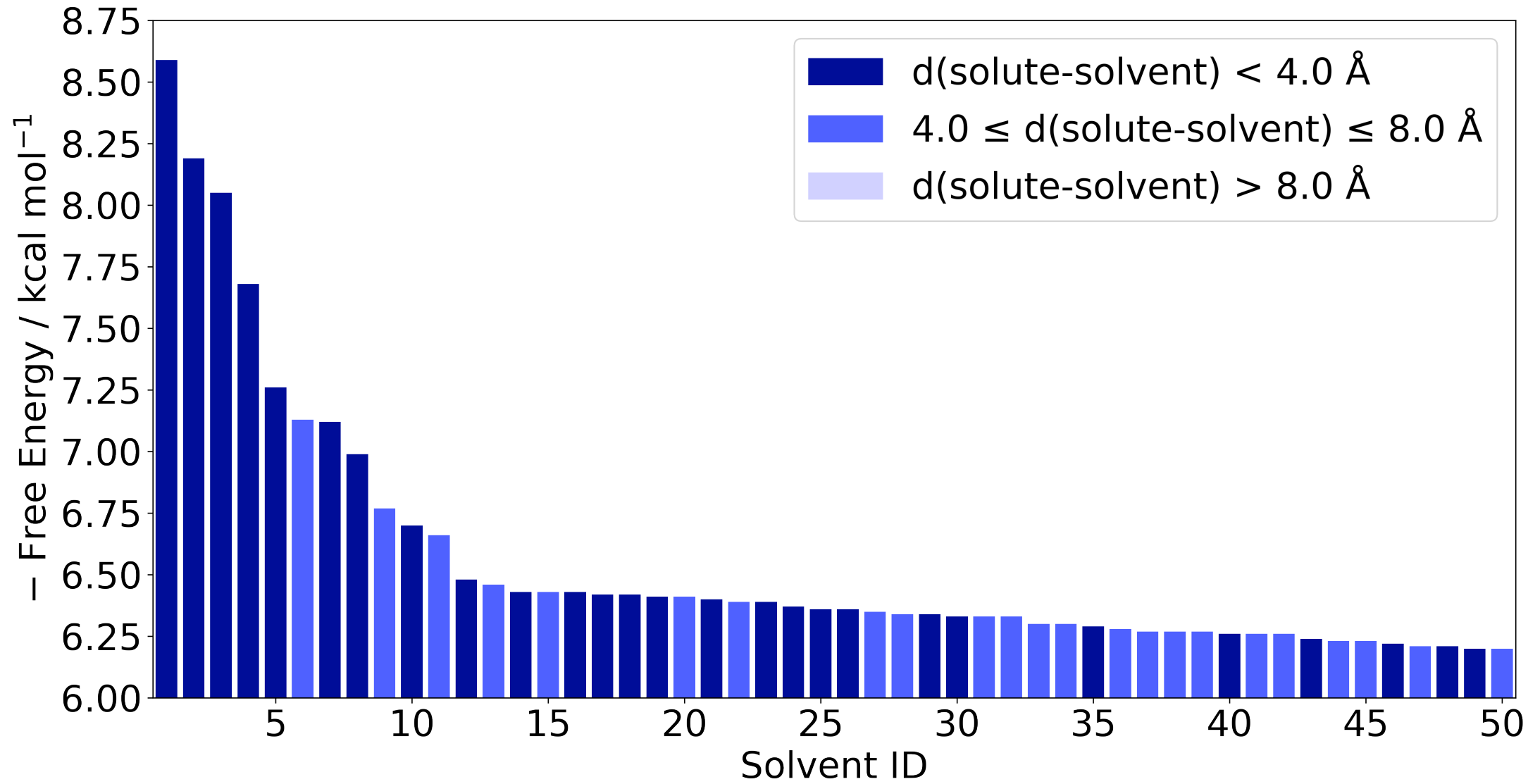

Figure S08: Resulting FEBISS 2 bar plot of the MoCat in DCM. The rapid decline in Free Energy towards the bulk value of -6.0 kcal mol^{-1} indicates extended areas of the solvent accessible surface of the catalyst which do not exhibit favorable solute-solvent interactions.

9 Predefined Solvents and Their Parameters

Table S02 provides an overview of the predefined solvents. The three letter shorthand notation is used as residue name of the solvent in the topology file. The given indices (P_1, P_0, P_2) point to solvent atoms used per default as the substructure for the determination of the solvent orientation. The index "0" indicates the first atom of the solvent. If - in the default case - the center of mass is used, P_0 is set to P_{-1} which FEBISS and also the modified CPPTRAJ version understands as COM.

The reference density and the reference value of E_{vv} were obtained from GIST simulations of the pure solvent, that is the solvent in itself.

First single solvent molecules were optimized using B3LYP/def2-TZVP/D4/C-PCM as implemented in ORCA 5.0.4. As a simulation force field GAFF was used utlizing RESP charges. A solvent box of roughly $60 \times 60 \times 60$ Å^3 was set up and filled with the respective solvent. The whole procedure, force field parametrization, simulation set up, simulation run, was streamlined using PyConSolv 1.0.6.3. A 100 ns restrained simulation comprising 10,000 frames was set up. The same settings as described in the main text were applied. The reference density was trivially obtained from the total number of solvent molecules divided by the average volume of the simulation box. gisttools of the Liedl group[10] was used to calculate E_{vv} of the neat unperturbed solvent from

$$E_{\mathrm{vv}}^{\mathrm{ref}} = V_{\mathrm{k}} N_{\mathrm{f}} \frac{\sum_{\mathrm{k}} E_{\mathrm{vv,unref}}^{\mathrm{dens}}}{\sum_{\mathrm{k}} N_{\mathrm{k}}}$$

where V_{k} is the voxel volume, N_{f} is the number of frames of the simulation, $E_{\mathrm{vv,unref}}^{\mathrm{dens}}$ the solvent-solvent energy value as obtained by the GIST simulation divided by the voxel volume, and N_{k} the number of solvent molecules that were registered in voxel k during the simulation.

The given atom indices used for determining the rigid substructure of the solvent molecule correspond to the indices of the predefined solvent molecules as given in Table S17 - S26 in the Appendix.

Table S02: Three letter abbreviation and the default atom indices of the solvent substructure used to calculate its orientation with respect to the lab coordinate system for solvents predefined in FEBISS 2. In addition, the reference density and $E_{\mathrm{vv,ref}}$ are given.

Solvent	Abbreviation	Atom indices (P_1, P_0, P_2)	ρ^0 [Å^{-3}]	$E_{\mathrm{vv,ref}}$ [kcal mol^{-1}molecule^{-1}]
Acetonitrile	ACN	3, 1, 0	0.0121	-11.51
Acetone	ACT	9, 1, 0	0.0085	-9.33
Benzene	BNZ	0, 1, 2	0.0066	-6.95
Chloroform	CL3	1, 0, 2	0.0073	-6.49
Tetrachloromethane	CL4	1, 0, 2	0.0061	-7.25
Dichloromethane	DCM	1, 0, 2	0.0094	-6.02
Dimethyl sulfoxide	DMS	9, 0, 1	0.0089	-14.77
Ethanol	ETL	0, 1, 7	0.0106	-12.8
Methanol	MTL	1, 4, 5	0.0154	-10.17
Water	WAT	1, 0, 2	0.0329	-9.544

References

(1) PodewitzLab https://github.com/PodewitzLab/FEBISS.

(2) Ong, S. P.; Richards, W. D.; Jain, A.; Hautier, G.; Kocher, M.; Cholia, S.; Gunter, D.; Chevrier, V. L.; Persson, K. A.; Ceder, G. Python Materials Genomics (pymatgen): A robust, open-source python library for materials analysis. *Comput. Mater. Sci* **2013**, *68*, 314–319, DOI: 10.1016/j.commatsci.2012.10.028.

(3) Togo, A.; Shinohara, K.; Tanaka, I. Spglib: a software library for crystal symmetry search. *Sci. Technol. Adv. Mater.: Methods* **2024**, *4*, 2384822, DOI: 10.1080/27660400.2024.2384822.

(4) Markley, F. L.; Cheng, Y.; Crassidis, J. L.; Oshman, Y. Averaging Quaternions. *J. Guid. Control Dyn.* **2007**, *30*, 1193–1197, DOI: 10.2514/1.28949.

(5) Hagen, C. https://github.com/christophhagen/averaging-quaternions.

(6) Roe, D. R.; Cheatham, T. E. I. PTRAJ and CPPTRAJ: Software for Processing and Analysis of Molecular Dynamics Trajectory Data. *J. Chem. Theory Comput.* **2013**, *9*, 3084–3095, DOI: 10.1021/ct400341p.

(7) Steiner, M.; Holzknecht, T.; Schauperl, M.; Podewitz, M. Quantum Chemical Microsolvation by Automated Water Placement. *Molecules* **2021**, *26*, Number: 6, 1793, DOI: 10.3390/molecules26061793.

(8) Waibl, F.; Kraml, J.; Hoerschinger, V. J.; Hofer, F.; Kamenik, A. S.; Fernández-Quintero, M. L.; Liedl, K. R. Grid inhomogeneous solvation theory for cross-solvation in rigid solvents. *J. Chem. Phys.* **2022**, *156*, 204101, DOI: 10.1063/5.0087549.

(9) Hu, M.; Zhang, Q.; Yang, J.; Li, X. Unit quaternion description of spatial rotations in 3D electron cryo-microscopy. *J. Struct. Biol.* **2020**, *212*, 107601, DOI: 10.1016/j.jsb.2020.107601.

(10) Liedllab https://github.com/liedllab/gisttools.

Appendix. Parameters of Solutes and Solvents

The tables S03 - S16 give the atom and bond parametrization of the solutes. The tables S17 - S26 give the coordinates of the predefined solvent molecules. The given indices (idx) correspond with the atom indices in Table S02 used to create the substructure as needed for the determination of the orientation.

Table S03: Atom parameters of the system BM-$CHCl_3$

#Atom	Name	Type	Charge	Mass	GBradius	rVDW	eVDW
1	C1	cc	0.3893	12.01	1.7	1.908	0.086
2	C2	cc	-0.3288	12.01	1.7	1.908	0.086
3	C3	cd	-0.1069	12.01	1.7	1.908	0.086
4	N4	na	0.288	14.01	1.55	1.824	0.17
5	C5	cd	-0.1489	12.01	1.7	1.908	0.086
6	C6	cc	-0.2759	12.01	1.7	1.908	0.086
7	H7	ha	0.1895	1.008	1.3	1.459	0.015
8	H8	h4	0.1817	1.008	1.3	1.409	0.015
9	H9	h4	0.1862	1.008	1.3	1.409	0.015
10	H10	ha	0.1891	1.008	1.3	1.459	0.015
11	C11	c3	-0.3453	12.01	1.7	1.908	0.1094
12	H12	h1	0.1447	1.008	1.3	1.387	0.0157
13	H13	h1	0.1447	1.008	1.3	1.387	0.0157
14	H14	h1	0.1447	1.008	1.3	1.387	0.0157
15	C15	cf	-0.4323	12.01	1.7	1.908	0.086
16	H16	ha	0.1781	1.008	1.3	1.459	0.015
17	C17	cf	-0.0913	12.01	1.7	1.908	0.086
18	H18	ha	0.1239	1.008	1.3	1.459	0.015
19	C19	cc	0.0768	12.01	1.7	1.908	0.086
20	C20	cc	-0.1967	12.01	1.7	1.908	0.086
21	C21	cc	-0.1	12.01	1.7	1.908	0.086
22	C22	cd	-0.3723	12.01	1.7	1.908	0.086
23	C23	cd	-0.4144	12.01	1.7	1.908	0.086
24	C24	c	0.7191	12.01	1.7	1.908	0.086
25	H25	ha	0.1572	1.008	1.3	1.459	0.015
26	H26	ha	0.1427	1.008	1.3	1.459	0.015
27	H27	ha	0.1497	1.008	1.3	1.459	0.015
28	H28	ha	0.1591	1.008	1.3	1.459	0.015
29	O29	o	-0.7515	16.0	1.5	1.6612	0.21

Table S04: Bond parameters of the system BM-$CHCl_3$

#Bnd	RK	REQ	A1	A2
1	349.1	1.084	2	7
2	352.0	1.082	3	8
3	352.0	1.082	5	9
4	349.1	1.084	6	10
5	330.6	1.097	11	12
6	330.6	1.097	11	13
7	330.6	1.097	11	14
8	342.5	1.088	15	16
9	342.5	1.088	17	18
10	349.1	1.084	20	25
11	349.1	1.084	21	26
12	349.1	1.084	22	27
13	349.1	1.084	23	28
14	419.8	1.428	1	2
15	419.8	1.428	1	6
16	513.0	1.366	1	15
17	500.9	1.373	2	3
18	425.8	1.38	3	4
19	425.8	1.38	4	5
20	327.7	1.463	4	11
21	500.9	1.373	5	6
22	382.8	1.457	15	17
23	513.0	1.366	17	19
24	419.8	1.428	19	20
25	419.8	1.428	19	21
26	500.9	1.373	20	22
27	500.9	1.373	21	23
28	371.0	1.468	22	24
29	371.0	1.468	23	24
30	637.7	1.218	24	29

Table S05: Atom parameters of the system BM-CCl_4

#Atom	Name	Type	Charge	Mass	GBradius	rVDW	eVDW
1	C1	cc	0.3849	12.01	1.7	1.908	0.086
2	C2	cc	-0.3283	12.01	1.7	1.908	0.086
3	C3	cd	-0.1278	12.01	1.7	1.908	0.086
4	N4	na	0.2858	14.01	1.55	1.824	0.17
5	C5	cd	-0.1737	12.01	1.7	1.908	0.086
6	C6	cc	-0.2671	12.01	1.7	1.908	0.086
7	H7	ha	0.1829	1.008	1.3	1.459	0.015
8	H8	h4	0.1744	1.008	1.3	1.409	0.015
9	H9	h4	0.1796	1.008	1.3	1.409	0.015
10	H10	ha	0.1805	1.008	1.3	1.459	0.015
11	C11	c3	-0.3395	12.01	1.7	1.908	0.1094
12	H12	h1	0.1367	1.008	1.3	1.387	0.0157
13	H13	h1	0.1367	1.008	1.3	1.387	0.0157
14	H14	h1	0.1367	1.008	1.3	1.387	0.0157
15	C15	cf	-0.4326	12.01	1.7	1.908	0.086
16	H16	ha	0.1745	1.008	1.3	1.459	0.015
17	C17	cf	-0.0884	12.01	1.7	1.908	0.086
18	H18	ha	0.1146	1.008	1.3	1.459	0.015
19	C19	cc	0.0905	12.01	1.7	1.908	0.086
20	C20	cc	-0.2009	12.01	1.7	1.908	0.086
21	C21	cc	-0.094	12.01	1.7	1.908	0.086
22	C22	cd	-0.3469	12.01	1.7	1.908	0.086
23	C23	cd	-0.3974	12.01	1.7	1.908	0.086
24	C24	c	0.7233	12.01	1.7	1.908	0.086
25	H25	ha	0.1516	1.008	1.3	1.459	0.015
26	H26	ha	0.1344	1.008	1.3	1.459	0.015
27	H27	ha	0.1473	1.008	1.3	1.459	0.015
28	H28	ha	0.1583	1.008	1.3	1.459	0.015
29	O29	o	-0.696	16.0	1.5	1.6612	0.21

Table S06: Bond parameters of the system BM-CCl_4

#Bnd	RK	REQ	A1	A2
1.0	349.1	1.084	2.0	7.0
2.0	352.0	1.082	3.0	8.0
3.0	352.0	1.082	5.0	9.0
4.0	349.1	1.084	6.0	10.0
5.0	330.6	1.097	11.0	12.0
6.0	330.6	1.097	11.0	13.0
7.0	330.6	1.097	11.0	14.0
8.0	342.5	1.088	15.0	16.0
9.0	342.5	1.088	17.0	18.0
10.0	349.1	1.084	20.0	25.0
11.0	349.1	1.084	21.0	26.0
12.0	349.1	1.084	22.0	27.0
13.0	349.1	1.084	23.0	28.0
14.0	419.8	1.428	1.0	2.0
15.0	419.8	1.428	1.0	6.0
16.0	513.0	1.366	1.0	15.0
17.0	500.9	1.373	2.0	3.0
18.0	425.8	1.38	3.0	4.0
19.0	425.8	1.38	4.0	5.0
20.0	327.7	1.463	4.0	11.0
21.0	500.9	1.373	5.0	6.0
22.0	382.8	1.457	15.0	17.0
23.0	513.0	1.366	17.0	19.0
24.0	419.8	1.428	19.0	20.0
25.0	419.8	1.428	19.0	21.0
26.0	500.9	1.373	20.0	22.0
27.0	500.9	1.373	21.0	23.0
28.0	371.0	1.468	22.0	24.0
29.0	371.0	1.468	23.0	24.0
30.0	637.7	1.218	24.0	29.0

Table S07: Atom parameters of the system BM-DMSO

#Atom	Name	Type	Charge	Mass	GBradius	rVDW	eVDW
1	C1	cc	0.3905	12.01	1.7	1.908	0.086
2	C2	cc	-0.3244	12.01	1.7	1.908	0.086
3	C3	cd	-0.083	12.01	1.7	1.908	0.086
4	N4	na	0.2894	14.01	1.55	1.824	0.17
5	C5	cd	-0.1209	12.01	1.7	1.908	0.086
6	C6	cc	-0.2799	12.01	1.7	1.908	0.086
7	H7	ha	0.195	1.008	1.3	1.459	0.015
8	H8	h4	0.188	1.008	1.3	1.409	0.015
9	H9	h4	0.1917	1.008	1.3	1.409	0.015
10	H10	ha	0.1961	1.008	1.3	1.459	0.015
11	C11	c3	-0.3509	12.01	1.7	1.908	0.1094
12	H12	h1	0.1524	1.008	1.3	1.387	0.0157
13	H13	h1	0.1524	1.008	1.3	1.387	0.0157
14	H14	h1	0.1524	1.008	1.3	1.387	0.0157
15	C15	cf	-0.4181	12.01	1.7	1.908	0.086
16	H16	ha	0.1791	1.008	1.3	1.459	0.015
17	C17	cf	-0.1038	12.01	1.7	1.908	0.086
18	H18	ha	0.1346	1.008	1.3	1.459	0.015
19	C19	cc	0.069	12.01	1.7	1.908	0.086
20	C20	cc	-0.1917	12.01	1.7	1.908	0.086
21	C21	cc	-0.109	12.01	1.7	1.908	0.086
22	C22	cd	-0.3975	12.01	1.7	1.908	0.086
23	C23	cd	-0.4297	12.01	1.7	1.908	0.086
24	C24	c	0.7065	12.01	1.7	1.908	0.086
25	H25	ha	0.1611	1.008	1.3	1.459	0.015
26	H26	ha	0.1506	1.008	1.3	1.459	0.015
27	H27	ha	0.1508	1.008	1.3	1.459	0.015
28	H28	ha	0.1583	1.008	1.3	1.459	0.015
29	O29	o	-0.8091	16.0	1.5	1.6612	0.21

Table S08: Bond parameters of the system BM-DMSO

#Bnd	RK	REQ	A1	A2
1	349.1	1.084	2	7
2	352.0	1.082	3	8
3	352.0	1.082	5	9
4	349.1	1.084	6	10
5	330.6	1.097	11	12
6	330.6	1.097	11	13
7	330.6	1.097	11	14
8	342.5	1.088	15	16
9	342.5	1.088	17	18
10	349.1	1.084	20	25
11	349.1	1.084	21	26
12	349.1	1.084	22	27
13	349.1	1.084	23	28
14	419.8	1.428	1	2
15	419.8	1.428	1	6
16	513.0	1.366	1	15
17	500.9	1.373	2	3
18	425.8	1.38	3	4
19	425.8	1.38	4	5
20	327.7	1.463	4	11
21	500.9	1.373	5	6
22	382.8	1.457	15	17
23	513.0	1.366	17	19
24	419.8	1.428	19	20
25	419.8	1.428	19	21
26	500.9	1.373	20	22
27	500.9	1.373	21	23
28	371.0	1.468	22	24
29	371.0	1.468	23	24
30	637.7	1.218	24	29

Table S09: Atom parameters of the system BM-MeOH

#Atom	Name	Type	Charge	Mass	GBradius	rVDW	eVDW
1	C1	cc	0.3905	12.01	1.7	1.908	0.086
2	C2	cc	-0.3249	12.01	1.7	1.908	0.086
3	C3	cd	-0.0844	12.01	1.7	1.908	0.086
4	N4	na	0.2894	14.01	1.55	1.824	0.17
5	C5	cd	-0.1227	12.01	1.7	1.908	0.086
6	C6	cc	-0.2797	12.01	1.7	1.908	0.086
7	H7	ha	0.1947	1.008	1.3	1.459	0.015
8	H8	h4	0.1877	1.008	1.3	1.409	0.015
9	H9	h4	0.1914	1.008	1.3	1.409	0.015
10	H10	ha	0.1957	1.008	1.3	1.459	0.015
11	C11	c3	-0.3507	12.01	1.7	1.908	0.1094
12	H12	h1	0.152	1.008	1.3	1.387	0.0157
13	H13	h1	0.152	1.008	1.3	1.387	0.0157
14	H14	h1	0.152	1.008	1.3	1.387	0.0157
15	C15	cf	-0.4191	12.01	1.7	1.908	0.086
16	H16	ha	0.1791	1.008	1.3	1.459	0.015
17	C17	cf	-0.1032	12.01	1.7	1.908	0.086
18	H18	ha	0.134	1.008	1.3	1.459	0.015
19	C19	cc	0.0698	12.01	1.7	1.908	0.086
20	C20	cc	-0.1925	12.01	1.7	1.908	0.086
21	C21	cc	-0.1089	12.01	1.7	1.908	0.086
22	C22	cd	-0.3954	12.01	1.7	1.908	0.086
23	C23	cd	-0.4283	12.01	1.7	1.908	0.086
24	C24	c	0.7067	12.01	1.7	1.908	0.086
25	H25	ha	0.161	1.008	1.3	1.459	0.015
26	H26	ha	0.1502	1.008	1.3	1.459	0.015
27	H27	ha	0.1506	1.008	1.3	1.459	0.015
28	H28	ha	0.1583	1.008	1.3	1.459	0.015
29	O29	o	-0.8055	16.0	1.5	1.6612	0.21

Table S10: Bond parameters of the system BM-MeOH

#Bnd	RK	REQ	A1	A2
1	349.1	1.084	2	7
2	352.0	1.082	3	8
3	352.0	1.082	5	9
4	349.1	1.084	6	10
5	330.6	1.097	11	12
6	330.6	1.097	11	13
7	330.6	1.097	11	14
8	342.5	1.088	15	16
9	342.5	1.088	17	18
10	349.1	1.084	20	25
11	349.1	1.084	21	26
12	349.1	1.084	22	27
13	349.1	1.084	23	28
14	419.8	1.428	1	2
15	419.8	1.428	1	6
16	513.0	1.366	1	15
17	500.9	1.373	2	3
18	425.8	1.38	3	4
19	425.8	1.38	4	5
20	327.7	1.463	4	11
21	500.9	1.373	5	6
22	382.8	1.457	15	17
23	513.0	1.366	17	19
24	419.8	1.428	19	20
25	419.8	1.428	19	21
26	500.9	1.373	20	22
27	500.9	1.373	21	23
28	371.0	1.468	22	24
29	371.0	1.468	23	24
30	637.7	1.218	24	29

Table S11: Atom parameters of the system Borneol-DMSO

#Atom	#Mol	Type	Charge	Mass	GBradius	rVDW	eVDW
1	C1	c3	-0.5361	12.01	1.7	1.908	0.1094
2	C2	c3	-0.5695	12.01	1.7	1.908	0.1094
3	C3	c3	-0.5324	12.01	1.7	1.908	0.1094
4	C4	c3	-0.0984	12.01	1.7	1.908	0.1094
5	C5	c3	-0.1005	12.01	1.7	1.908	0.1094
6	C6	c3	-0.2289	12.01	1.7	1.908	0.1094
7	C7	c3	0.0346	12.01	1.7	1.908	0.1094
8	C8	c3	0.4043	12.01	1.7	1.908	0.1094
9	C9	c3	0.5119	12.01	1.7	1.908	0.1094
10	C10	c3	0.3725	12.01	1.7	1.908	0.1094
11	O11	oh	-0.7513	16.0	1.5	1.721	0.2104
12	H12	hc	0.1077	1.008	1.3	1.487	0.0157
13	H13	hc	0.1077	1.008	1.3	1.487	0.0157
14	H14	hc	0.1077	1.008	1.3	1.487	0.0157
15	H15	hc	0.1216	1.008	1.3	1.487	0.0157
16	H16	hc	0.1216	1.008	1.3	1.487	0.0157
17	H17	hc	0.1216	1.008	1.3	1.487	0.0157
18	H18	hc	0.1038	1.008	1.3	1.487	0.0157
19	H19	hc	0.1038	1.008	1.3	1.487	0.0157
20	H20	hc	0.1038	1.008	1.3	1.487	0.0157
21	H21	hc	0.0244	1.008	1.3	1.487	0.0157
22	H22	hc	0.0244	1.008	1.3	1.487	0.0157
23	H23	hc	0.0033	1.008	1.3	1.487	0.0157
24	H24	hc	0.0033	1.008	1.3	1.487	0.0157
25	H25	hc	0.0583	1.008	1.3	1.487	0.0157
26	H26	hc	0.0583	1.008	1.3	1.487	0.0157
27	H27	hc	-0.0331	1.008	1.3	1.487	0.0157
28	H28	h1	-0.073	1.008	1.3	1.387	0.0157
29	H29	ho	0.4285	1.008	0.8	0.0	0.0

Table S12: Bond parameters of the system Borneol-DMSO

#Bnd	RK	REQ	A1	A2
1	330.6	1.097	1	12
2	330.6	1.097	1	13
3	330.6	1.097	1	14
4	330.6	1.097	2	15
5	330.6	1.097	2	16
6	330.6	1.097	2	17
7	330.6	1.097	3	18
8	330.6	1.097	3	19
9	330.6	1.097	3	20
10	330.6	1.097	4	21
11	330.6	1.097	4	22
12	330.6	1.097	5	23
13	330.6	1.097	5	24
14	330.6	1.097	6	25
15	330.6	1.097	6	26
16	330.6	1.097	7	27
17	330.6	1.097	8	28
18	371.4	0.973	11	29
19	300.9	1.538	1	9
20	300.9	1.538	2	9
21	300.9	1.538	3	10
22	300.9	1.538	4	5
23	300.9	1.538	4	7
24	300.9	1.538	5	10
25	300.9	1.538	6	7
26	300.9	1.538	6	8
27	300.9	1.538	7	9
28	300.9	1.538	8	10
29	316.7	1.423	8	11
30	300.9	1.538	9	10

Table S13: Atom parameters of the system MoCat-DCM

#Atom	Name	Type	Charge	Mass	GBradius	rVDW	eVDW
1	MO	M1	1.4484	95.96	1.5	1.506	0.056
2	N1	Y4	-0.5883	14.01	1.55	1.824	0.17
3	C2	ca	-0.0542	12.01	1.7	1.908	0.086
4	C3	ca	0.2182	12.01	1.7	1.908	0.086
5	C4	ca	-0.3231	12.01	1.7	1.908	0.086
6	H5	ha	0.1687	1.008	1.3	1.459	0.015
7	C6	ca	-0.0231	12.01	1.7	1.908	0.086
8	H7	ha	0.1305	1.008	1.3	1.459	0.015
9	C8	ca	-0.3588	12.01	1.7	1.908	0.086
10	H9	ha	0.1703	1.008	1.3	1.459	0.015
11	C10	ca	0.3254	12.01	1.7	1.908	0.086
12	C11	c3	-0.1859	12.01	1.7	1.908	0.1094
13	H12	hc	0.0702	1.008	1.3	1.487	0.0157
14	H13	hc	0.0702	1.008	1.3	1.487	0.0157
15	H14	hc	0.0702	1.008	1.3	1.487	0.0157
16	C15	c3	-0.2624	12.01	1.7	1.908	0.1094
17	H16	hc	0.0884	1.008	1.3	1.487	0.0157
18	H17	hc	0.0884	1.008	1.3	1.487	0.0157
19	H18	hc	0.0884	1.008	1.3	1.487	0.0157
20	C19	Y5	-0.2918	12.01	1.7	1.908	0.21
21	N20	nh	0.2281	14.01	1.55	1.824	0.17
22	C21	c2	-0.1422	12.01	1.7	1.908	0.086
23	H22	h4	0.2085	1.008	1.3	1.409	0.015
24	N23	nh	0.4071	14.01	1.55	1.824	0.17
25	C24	c2	-0.2629	12.01	1.7	1.908	0.086
26	H25	h4	0.2241	1.008	1.3	1.409	0.015
27	C26	ca	-0.254	12.01	1.7	1.908	0.086
28	C27	ca	0.1939	12.01	1.7	1.908	0.086
29	C28	ca	-0.423	12.01	1.7	1.908	0.086
30	H29	ha	0.1896	1.008	1.3	1.459	0.015
31	C30	ca	0.3267	12.01	1.7	1.908	0.086
32	C31	ca	-0.4345	12.01	1.7	1.908	0.086
33	H32	ha	0.1946	1.008	1.3	1.459	0.015
34	C33	ca	0.2184	12.01	1.7	1.908	0.086
35	C34	c3	-0.1068	12.01	1.7	1.908	0.1094
36	H35	hc	0.0373	1.008	1.3	1.487	0.0157
37	H36	hc	0.0373	1.008	1.3	1.487	0.0157
38	H37	hc	0.0373	1.008	1.3	1.487	0.0157
39	C38	c3	-0.2433	12.01	1.7	1.908	0.1094
40	H39	hc	0.0794	1.008	1.3	1.487	0.0157

Table S13 (cont.): Atom parameters of the system MoCat-DCM

#Atom	Name	Type	Charge	Mass	GBradius	rVDW	eVDW
41	H40	hc	0.0794	1.008	1.3	1.487	0.0157
42	H41	hc	0.0794	1.008	1.3	1.487	0.0157
43	C42	c3	-0.2512	12.01	1.7	1.908	0.1094
44	H43	hc	0.0878	1.008	1.3	1.487	0.0157
45	H44	hc	0.0878	1.008	1.3	1.487	0.0157
46	H45	hc	0.0878	1.008	1.3	1.487	0.0157
47	C46	ca	-0.2871	12.01	1.7	1.908	0.086
48	C47	ca	0.2151	12.01	1.7	1.908	0.086
49	C48	ca	-0.3938	12.01	1.7	1.908	0.086
50	H49	ha	0.1846	1.008	1.3	1.459	0.015
51	C50	ca	0.2764	12.01	1.7	1.908	0.086
52	C51	ca	-0.3882	12.01	1.7	1.908	0.086
53	H52	ha	0.1862	1.008	1.3	1.459	0.015
54	C53	ca	0.1618	12.01	1.7	1.908	0.086
55	C54	c3	-0.2823	12.01	1.7	1.908	0.1094
56	H55	hc	0.0979	1.008	1.3	1.487	0.0157
57	H56	hc	0.0979	1.008	1.3	1.487	0.0157
58	H57	hc	0.0979	1.008	1.3	1.487	0.0157
59	C58	c3	-0.2031	12.01	1.7	1.908	0.1094
60	H59	hc	0.0705	1.008	1.3	1.487	0.0157
61	H60	hc	0.0705	1.008	1.3	1.487	0.0157
62	H61	hc	0.0705	1.008	1.3	1.487	0.0157
63	C62	c3	-0.0842	12.01	1.7	1.908	0.1094
64	H63	hc	0.0355	1.008	1.3	1.487	0.0157
65	H64	hc	0.0355	1.008	1.3	1.487	0.0157
66	H65	hc	0.0355	1.008	1.3	1.487	0.0157
67	C66	Y6	-1.0795	12.01	1.7	1.908	0.21
68	H67	ha	0.3265	1.008	1.2	1.459	0.015
69	C68	c3	0.6621	12.01	1.7	1.908	0.1094
70	C69	c3	-0.3794	12.01	1.7	1.908	0.1094
71	H70	hc	0.0886	1.008	1.3	1.487	0.0157
72	H71	hc	0.0886	1.008	1.3	1.487	0.0157
73	H72	hc	0.0886	1.008	1.3	1.487	0.0157
74	C73	c3	-0.234	12.01	1.7	1.908	0.1094
75	H74	hc	0.0522	1.008	1.3	1.487	0.0157
76	H75	hc	0.0522	1.008	1.3	1.487	0.0157
77	H76	hc	0.0522	1.008	1.3	1.487	0.0157
78	C77	c3	-0.3377	12.01	1.7	1.908	0.1094
79	H78	hc	0.0862	1.008	1.3	1.487	0.0157
80	H79	hc	0.0862	1.008	1.3	1.487	0.0157

Table S13 (cont.): Atom parameters of the system MoCat-DCM

#Atom	Name	Type	Charge	Mass	GBradius	rVDW	eVDW
81	H80	hc	0.0862	1.008	1.3	1.487	0.0157
82	S81	s6	0.8262	32.06	1.8	2.0	0.25
83	O82	Y7	-0.565	16.0	1.5	1.6612	0.21
84	O83	o	-0.4816	16.0	1.5	1.6612	0.21
85	O84	o	-0.4503	16.0	1.5	1.6612	0.21
86	C85	c3	0.4916	12.01	1.7	1.908	0.1094
87	F86	f	-0.1543	19.0	1.5	1.75	0.061
88	F87	f	-0.1369	19.0	1.5	1.75	0.061
89	F88	f	-0.1617	19.0	1.5	1.75	0.061
90	S89	s6	0.7585	32.06	1.8	2.0	0.25
91	O90	Y8	-0.415	16.0	1.5	1.6612	0.21
92	O91	o	-0.445	16.0	1.5	1.6612	0.21
93	O92	o	-0.4657	16.0	1.5	1.6612	0.21
94	C93	c3	0.4538	12.01	1.7	1.908	0.1094
95	F94	f	-0.1312	19.0	1.5	1.75	0.061
96	F95	f	-0.1501	19.0	1.5	1.75	0.061
97	F96	f	-0.1453	19.0	1.5	1.75	0.061

Table S14: Bond parameters of the system MoCat-DCM

#Bnd	RK	REQ	A1	A2
1	330.6	1.097	16	17
2	330.6	1.097	16	18
3	330.6	1.097	16	19
4	330.6	1.097	12	13
5	330.6	1.097	12	14
6	330.6	1.097	12	15
7	345.8	1.086	9	10
8	345.8	1.086	7	8
9	345.8	1.086	5	6
10	330.6	1.097	63	64
11	330.6	1.097	63	65
12	330.6	1.097	63	66
13	330.6	1.097	59	60
14	330.6	1.097	59	61
15	330.6	1.097	59	62
16	330.6	1.097	55	56
17	330.6	1.097	55	57
18	330.6	1.097	55	58
19	345.8	1.086	52	53
20	345.8	1.086	49	50
21	330.6	1.097	43	44
22	330.6	1.097	43	45
23	330.6	1.097	43	46
24	330.6	1.097	39	40
25	330.6	1.097	39	41
26	330.6	1.097	39	42
27	330.6	1.097	35	36
28	330.6	1.097	35	37
29	330.6	1.097	35	38
30	345.8	1.086	32	33
31	345.8	1.086	29	30
32	344.6	1.087	25	26
33	344.6	1.087	22	23
34	330.6	1.097	78	79
35	330.6	1.097	78	80
36	330.6	1.097	78	81
37	330.6	1.097	74	75
38	330.6	1.097	74	76
39	330.6	1.097	74	77
40	330.6	1.097	70	71

Table S14 (cont.): Bond parameters of the system MoCat-DCM

#Bnd	RK	REQ	A1	A2
41	330.6	1.097	70	72
42	330.6	1.097	70	73
43	374.7	1.067	67	68
44	409.9	1.748	1	2
45	100.5	2.181	1	20
46	300.4	1.876	1	67
47	54.1	2.107	1	83
48	52.8	2.113	1	91
49	321.0	1.516	11	16
50	461.1	1.398	9	11
51	461.1	1.398	7	9
52	461.1	1.398	5	7
53	461.1	1.398	4	5
54	321.0	1.516	4	12
55	461.1	1.398	3	4
56	461.1	1.398	3	11
57	494.6	1.335	2	3
58	321.0	1.516	54	63
59	461.1	1.398	52	54
60	461.1	1.398	51	52
61	321.0	1.516	51	59
62	461.1	1.398	49	51
63	461.1	1.398	48	49
64	321.0	1.516	48	55
65	461.1	1.398	47	48
66	461.1	1.398	47	54
67	321.0	1.516	34	43
68	461.1	1.398	32	34
69	461.1	1.398	31	32
70	321.0	1.516	31	39
71	461.1	1.398	29	31
72	461.1	1.398	28	29
73	321.0	1.516	28	35
74	461.1	1.398	27	28
75	461.1	1.398	27	34
76	416.2	1.387	24	25
77	417.9	1.386	24	47
78	569.4	1.334	22	25
79	416.2	1.387	21	22
80	417.9	1.386	21	27

Table S14 (cont.): Bond parameters of the system MoCat-DCM

#Bnd	RK	REQ	A1	A2
81	482.6	1.342	20	21
82	482.6	1.342	20	24
83	300.9	1.538	69	70
84	300.9	1.538	69	74
85	300.9	1.538	69	78
86	371.6	1.467	67	69
87	356.9	1.35	86	87
88	356.9	1.35	86	88
89	356.9	1.35	86	89
90	512.7	1.453	82	83
91	512.7	1.453	82	84
92	512.7	1.453	82	85
93	233.5	1.808	82	86
94	356.9	1.35	94	95
95	356.9	1.35	94	96
96	356.9	1.35	94	97
97	512.7	1.453	90	91
98	512.7	1.453	90	92
99	512.7	1.453	90	93
100	233.5	1.808	90	94

Table S15: Atom parameters of the system Urea-H_2O

#Atom	Name	Type	Charge	Mass	GBradius	rVDW	eVDW
1	H1	hn	0.4553	1.008	1.3	0.6	0.0157
2	O2	o	-0.7494	16.0	1.5	1.6612	0.21
3	C3	c	0.9989	12.01	1.7	1.908	0.086
4	N4	n	-1.0136	14.01	1.55	1.824	0.17
5	H5	hn	0.4335	1.008	1.3	0.6	0.0157
6	N6	n	-1.0115	14.01	1.55	1.824	0.17
7	H7	hn	0.4547	1.008	1.3	0.6	0.0157
8	H8	hn	0.4321	1.008	1.3	0.6	0.0157

Table S16: Bond parameters of the system Urea-H_2O

#Bnd	RK	REQ	A1	A2
1	403.2	1.013	1	4
2	403.2	1.013	4	5
3	403.2	1.013	6	7
4	403.2	1.013	6	8
5	637.7	1.218	2	3
6	427.6	1.379	3	4
7	427.6	1.379	3	6

Table S17: Indices and coordinates of the predefined acetonitrile molecule

idx	Name	X	Y	Z
0	C	-5.94	0.917	0.191
1	C1	-4.493	0.864	0.045
2	N1	-7.09	0.958	0.307
3	H1	-4.216	0.002	-0.559
4	H2	-4.028	0.78	1.025
5	H3	-4.14	1.771	-0.443

Table S18: Indices and coordinates of the predefined acetone molecule

idx	Name	X	Y	Z
0	C	-7.583	-1.817	0.042
1	C1	-6.096	-1.815	0.281
2	H	-8.043	-1.001	0.603
3	H1	-8.02	-2.745	0.414
4	H2	-7.798	-1.703	-1.017
5	C2	-5.642	-2.05	1.697
6	H3	-6.109	-1.325	2.365
7	H4	-4.56	-1.983	1.767
8	H5	-5.972	-3.039	2.022
9	O	-5.301	-1.629	-0.626

Table S19: Indices and coordinates of the predefined benzene molecule

idx	Name	X	Y	Z
0	C	-7.995	-1.854	-1.927
1	C1	-6.609	-1.797	-2.063
2	C2	-5.821	-1.372	-0.996
3	H	-6.145	-2.083	-2.998
4	C3	-6.419	-1.003	0.208
5	H1	-4.745	-1.327	-1.102
6	C4	-7.804	-1.059	0.344
7	H2	-5.807	-0.672	1.037
8	H3	-8.268	-0.772	1.278
9	C5	-8.592	-1.485	-0.724
10	H4	-9.669	-1.528	-0.618
11	H5	-8.607	-2.184	-2.756

Table S20: Indices and coordinates of the predefined chloroform molecule

idx	Name	X	Y	Z
0	C	-6.012	-1.302	-1.671
1	Cl	-4.24	-1.266	-1.698
2	H	-6.372	-0.482	-2.278
3	Cl1	-6.624	-1.076	-0.023
4	Cl2	-6.623	-2.812	-2.369

Table S21: Indices and coordinates of the predefined tetrachloromethane molecule

idx	Name	X	Y	Z
0	C	-5.974	-1.387	-1.608
1	Cl	-4.199	-1.387	-1.608
2	Cl1	-6.566	-0.042	-2.603
3	Cl2	-6.566	-1.198	0.055
4	Cl3	-6.566	-2.922	-2.275

Table S22: Indices and coordinates of the predefined dichloromethane molecule

idx	Name	X	Y	Z
0	C	-6.065	-1.27	-1.557
1	Cl	-4.286	-1.233	-1.541
2	H	-6.412	-0.461	-2.185
3	H1	-6.412	-1.178	-0.536
4	Cl1	-6.696	-2.796	-2.22

Table S23: Indices and coordinates of the predefined dimethyl sulfoxide molecule

idx	Name	X	Y	Z
0	S	-6.258	-4.64	-0.038
1	C	-5.594	-2.98	0.172
2	C1	-5.346	-5.416	1.306
3	H	-5.587	-6.477	1.281
4	H1	-5.67	-4.983	2.252
5	H2	-4.28	-5.262	1.148
6	H3	-6.007	-2.369	-0.628
7	H4	-4.508	-3.02	0.102
8	H5	-5.912	-2.591	1.139
9	O	-5.677	-5.178	-1.318

Table S24: Indices and coordinates of the predefined ethanol molecule

idx	Name	X	Y	Z
0	C	-5.835	2.868	-0.053
1	C1	-4.319	2.919	-0.052
2	H	-6.191	2.202	-0.841
3	H1	-6.254	3.86	-0.234
4	H2	-6.209	2.503	0.904
5	H3	-3.951	3.258	-1.025
6	H4	-3.902	1.931	0.136
7	O	-3.8	3.757	0.987
8	H5	-4.121	4.653	0.83

Table S25: Indices and coordinates of the predefined methanol molecule

idx	Name	X	Y	Z
0	H	-5.472	2.903	-0.037
1	C	-4.38	2.922	-0.051
2	H1	-4.042	3.245	-1.038
3	H2	-4.011	1.915	0.131
4	O	-3.854	3.751	0.984
5	H3	-4.168	4.65	0.833

Table S26: Indices and coordinates of the predefined water molecule

idx	Name	X	Y	Z
0	O	0.0	0.0	0.0
1	H	0.6231	0.6757	-0.2671
2	H1	-0.6116	0.4525	0.5809
